# Prevalence of Sleep Disturbance in Patients With Cancer: A Systematic Review and Meta-Analysis

**DOI:** 10.1177/10547738221092146

**Published:** 2022-04-28

**Authors:** Mohammed Al Maqbali, Mohammed Al Sinani, Ahmad Alsayed, Alexander M. Gleason

**Affiliations:** 1 Northumbria University, Newcastle-Upon-Tyne, UK; 2 Imperial College London, London, UK; 3 Ministry of Health, Al Buraimi, Oman; 4 Applied Science Private University, Amman, Jorden; 5 Fatima College of Health Sciences, Abu Dhabi, UAE

**Keywords:** prevalence, sleep disturbance, insomnia, cancer, meta-analysis

## Abstract

Sleep disturbance is one of the most common and troubling symptoms that harm the quality of life throughout all phases of treatment and stages of the illness among patients with cancer. The aim of this meta-analysis is to examine the present status of sleep disturbance prevalence in patients with cancer. The following databases were searched: PubMed, CINAHL, MEDLINE, EMBASE, PsycINFO, from inception to April 2021. Prevalence rates were pooled with meta-analysis using a random-effects model. A total of 160 studies (*N* = 46,279) published between 1998 and 2021 met the inclusion criteria. The overall prevalence of sleep disturbance was 60.7% (26,448/46,279 participants, 95% CI 58.1–63.3%) with significant heterogeneity between studies (*p* < .000, *τ*^2^ = .0000, *I*^2^ = 96.4%). This meta-analysis highlights the importance of developing optimal monitoring strategies to reduce sleep disturbance and improve the quality of life of cancer patients.

## Introduction

A diagnosis of cancer is a significant life stressor that can affect the physiological, psychological, and physical state of the individuals. One of the most common and distressing symptoms is sleep disturbance, which negatively affects the quality of life among patients diagnosed with cancer. The World Health Organization estimates there were 19.3 million cases of new cancers diagnosed and almost 10 million deaths in 2020; moreover, the number of new cancers diagnoses worldwide is expected to rise in the next two decades by about 47% (Sung et al., 2021).

There are potential difficulties in defining sleep disturbance or disorder, as sleep quality is a multidimensional concept. Buysse (2014) defines sleep health as ‘a multidimensional pattern of sleep-wakefulness, adapted to individual, social, and environmental demands, that promotes physical and mental well-being. Specifically, sleep disturbance may include difficulty falling asleep, problems with the initiation and maintenance of sleep, poor sleep timing, quality, efficiency, and excessive daytime sleepiness (Berger et al., 2018; Otte et al., 2016).

Evidence of sleep disturbance in cancer patients can have a negative impact on health-related quality of life, which includes physical and psychological functioning (Palesh et al., 2010; Rolke et al., 2009). Sleep disturbance has also been linked with the likelihood of cancer recurring (Sigurdardottir et al., 2013) and may result in poor healing (Otte et al., 2015), decreased cognitive functioning (Schagen et al., 2014), and reduced work activity (Die Trill, 2013).

Although the prevalence of sleep disturbance has been extensively studied in patients diagnosed with cancer, healthcare professionals often underestimate the importance of such symptoms (Laugsand et al., 2010). There is a lack of information related to the prevalence of sleep disturbance and its effect on patients in terms of whether or not it has a negative impact on their quality of life (Al Maqbali et al., 2021; Fox et al., 2020) and is associated with high levels of disability (Lourenço et al., 2020). This review will be useful for providing precise estimates of the prevalence of sleep disturbance and identifying the potential risk factors that may affect reported prevalence rates. In addition, it will provide evidence-based recommendations for sleep disturbance in terms of ensuring increased awareness, better control and treatment, and better nursing management for sleep disturbance.

Two previous systematic reviews and meta-analyses have been published to determine the prevalence of sleep disturbance among different type of patients with cancer (Leysen et al., 2019; Santoso et al., 2019). Whilst these reviews are very helpful, they focused on a single type of cancer, mainly head and neck (Santoso et al., 2019) and breast cancer (Leysen et al., 2019). Therefore, different types of patients were not included. Although the risk of bias was assessed coarsely as a part of previous systematic reviews and meta-analyses, none of the reviews has attempted to examine the publication bias of the included studies. Hence, the current meta-analysis was designed and conducted to estimate the raw and weighted prevalence rates of sleep disturbances among cancer patients, taking into account the effect of a single moderator and the simultaneous interactions of several moderators, on the prevalence of sleep disturbance. Knowledge of the prevalence of sleep disturbance among cancer patients is extremely important in the assessment, treatment and management of patients, staff education, and further assessments required for the healthcare system.

This meta-analysis is significant for several reasons. First, a precise, reliable, and valid estimate of just how prevalent sleep disturbance is among cancer patients can support the need for more emphasis on sleep in degree and continuing education programs, as well as provide strong evidence for implementing general policies and procedures that promote sleep in cancer care settings. Second, identifying the factors affecting sleep disturbance through subgroup analyses, provides a solid foundation for further research that could help to inform intervention for patient assessment, treatment, and the management of sleep disturbance.

## Methods

This systematic review and meta-analysis was undertaken according to the PRISMA standards (Page et al., 2021).

### Search Strategy

A systematic literature search, between October 1976 and 10 April 2021, was conducted using the following databases: PubMed, CINHAL, MEDLINE, EMBASE, PsycINFO, Cochrane Library. Search terms used both free text words and medical subject headings, that is, MeSH terms, to search papers in the review; that is, (MH “Sleep*”) OR (MH “ Insomnia”) OR (MH “sleep disturbances”) OR (MH “sleep disorder”) OR (MH “Narcolepsy”) OR (MH “Sleep Apnea*”) OR (MH “Circadian Rhythm”) OR (MH “Sleep Wake Transition Disorders”) OR (MH “Sleep Apnea, Obstructive”) OR (“ sleep disordered breathing “) AND (MH “Neoplasms+”) OR “neoplasm*” OR “tumour*” OR “tumor*” OR “cancer*” OR “Hodgkin*” OR “haematolog*” OR “radiat*” OR “radioth*” OR “chemo*” OR (MH “Hormone Therapy+”) OR “bone marrow transplant*” AND (MH “Epidemiology+”) OR (MH “Epidemiological Research+”) OR (MH “Incident Reports+”) OR “prevalence or incidence or statistics” OR (MH “Prevalence”) OR (MH “Surveys+”) OR (MH “Prospective Studies+”) OR (MH “Cross Sectional+”) OR (MH “Longitudinal+”). In addition, reference lists were screened of the retrieved studies and reviewer articles to identify any further studies.

### Study Selection

Two investigators (MM and SM) performed the search, scrutinizing all titles and abstracts for eligibility against the inclusion and exclusion criteria. Any disagreements were resolved by discussion with a third investigator (BK). Studies were included in the review according to the following inclusion criteria: (1) reported prevalence of sleep disturbance; (2) diagnosed with any type of cancer; (3) subjects were aged 18 or older; (4) cross-sectional or cohort survey (only the baseline data were extracted); (5) sample size more than 50 to avoid selection bias from small studies; (6) studies published in English in a peer-reviewed journal. The exclusion criteria were: (1) protocol papers and conference abstracts; (2) if sleep disturbance was assessed used single question; (3) study included non-cancer and cancer participants; (4) not published in English.

### Quality Assessment

Upon retrieval of applicable studies, the quality assessment was completed using the Newcastle-Ottawa Scale (NOS) (Well et al., 2020). This scale consists of eight items that evaluate the non-randomized studies, which covered three criteria: the selection of the participants, comparability of study groups, and outcome assessment. The NOS uses a scoring system with the lowest possible score of zero and the highest possible score of nine. The total points awarded indicate the overall quality of the study. A study was determined to be of low risk of bias when the score was 7 to 9, of moderate risk of bias if the score was 5 to 6, and high risk of bias if the score was 0 to 4 (Li & Katikireddi, 2019).

### Data Analyses

The mean point of sleep disturbance prevalence, odds ratios (ORs) with 95% confidence interval was calculated as effect size by using a random-effects model. Heterogeneity was tested using *I*-squared (*I*^2^) statistics. A value of *I*^2^ was considered to be low with 0 to 25%, 25 to 50% as moderate, and 50 to 75% considered to be high heterogeneity (Higgins et al., 2003). In addition, subgroup analyses to test the significant differences in the prevalence of sleep disturbance between different groups (age, publication year, continent, NOS, study design, type of cancer, stage of cancer, treatment status, and scale used) the analyses were performed when at least have four studies per subgroup. A sensitivity analysis was performed by removing one study at a time to evaluate the impact of pooled prevalence of remaining studies (Patsopoulos et al., 2008).

Meta-regression analyses were performed for moderating continuous variables (mean age and female percentage) (Higgins et al., 2019). Funnel plots were found to be an inaccurate method for assessing publication bias in meta-analyses of proportion studies (Hunter et al., 2014; Moreno et al., 2009). Therefore, publication bias was estimated using Egger’s linear regression test and funnel plot (Egger et al., 1997). If there is a significant publication bias, the Trim and Fill method was adapted to assess the robustness of the results (Duval & Tweedie, 2000). A *p*-value of less than .05 was considered statistically significant. Meta-analysis was conducted using the Comprehensive Meta-Analysis software, version 2.2 (Biostat, Englewood, NJ, USA). Forest plots were constructed using a Microsoft Excel spreadsheet constructed by Neyeloff et al. (2012).

## Results

**Figure 1.**
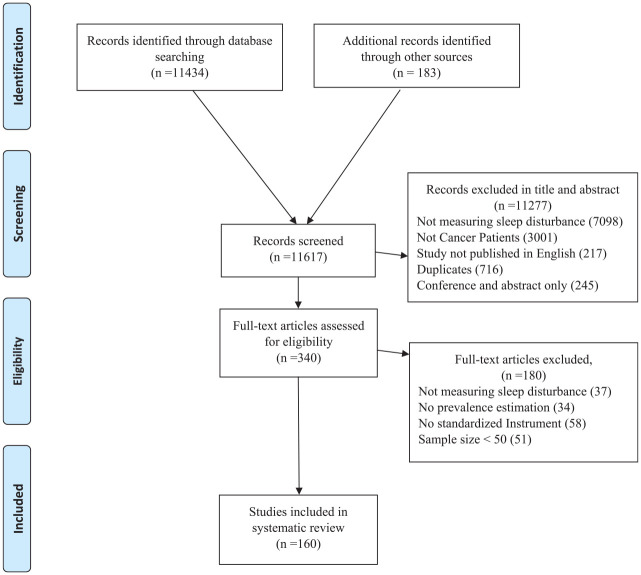
PRISMA diagram.

The database search identified 11,434 papers; these were screened by abstract and title (Figure 1 shows the PRISMA flow chart). Of these, 11,277 papers were excluded during the title and abstract screening for the following reasons: 7,098 papers did not measure sleep disturbance; 3,001 did not include patients with cancer; 716 were duplicated papers; 245 were conference, and abstract papers, and 217 were not in English. One hundred eighty papers were excluded during full-text review. As such, 159 studies were identified as eligible for meta-analysis. However, one study (Savard et al., 2015) reported prevalence of sleep disturbance for two different types of cancer, each type of cancer was evaluated separately. For this double reporting in one study, the interpretation of the studies included in this meta-analysis reported had 160 studies instead of 159.

### General Characteristics

One hundred sixty studies involving 46,279 mixed cancer patients were included in this meta-analysis (males = 11,574; females = 34,705). All studies were published between 1998 and 2021. The vast majority (*n* = 64 studies) included patients with breast cancer, followed by studies with mixed cancer diagnoses (*n* = 57). Seventy-one studies reported sleep disturbance levels on mixed stages of cancer; however, 80 studies reported on patients undergoing mixed cancer treatment. Fifty-two studies reported patients completed more than 3-month post-treatment. This meta-analysis included 96 cross-sectional and 64 longitudinal design studies.

More than half of the studies used Pittsburgh Sleep Quality Index (PSQI) scale (*n* = 96), followed by 31 studies that used Insomnia Severity Index (ISI). Seventy-five studies originated from the United States, twelve from Canada, eight from each Iran and China, six from Taiwan, five from Korea, four from each Australia, Greek, Italy, Japan, and Turkey, three from each Germany, and Norway, two from each Brazil, Hong Kong, Oman, and the UK, and one from each of the following: Belgium, Denmark, France, India, Morocco, Netherland, Pakistan, Portugal, Switzerland, Taiwan, Tunis, and Vietnam (See Supplementary Table 1 for a General Characteristics of Studies).

### Quality Assessment

The studies were assessed using the NOS checklist. Thirty studies were classified as having a low risk of bias and 130 as moderate. The detailed results of the quality assessment of studies included in this meta-analysis are listed in Supplementary Table 2.

### Prevalence of Sleep Disturbance

**Figure 2.**
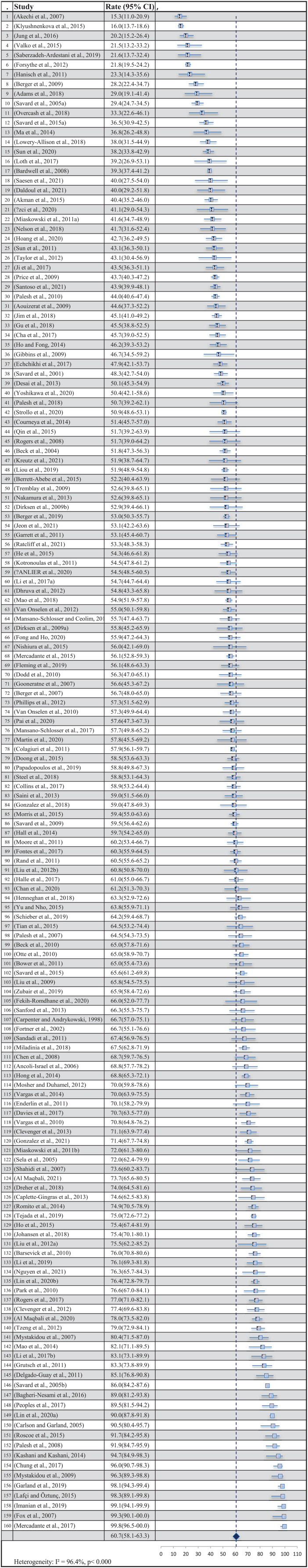
Forest plot of the prevalence of sleep disturbance.

The overall pooled point estimates of prevalence for sleep disturbance varied between 15.3% (Akechi et al., 2007) and 99.8% (Mercadante et al., 2017) (Figure 2: Forest plots). All meta-analyses of prevalence of estimates of sleep disturbance reported by the 160 studies yielded a summary prevalence of 60.7% (26,448/46,279 participants, 95% CI 58.1–63.3). Sensitivity analysis by excluding one study each time demonstrated that no differences in the overall estimation by more than 1%. There was significant heterogeneity between studies when it came to estimating the prevalence in terms of sleep disturbance (*p* < .000, *τ*^2^ = 0.0000, *I*^2^ = 96.4%).

Patients with cancer who were between 40 and 49 years old showed the highest sleep disturbance prevalence at 67% (95% CI = 65.3–76.2, *I*^2^ = 93.6), whereas the lowest sleep disturbance prevalence was reported in the 50 to 59 age group at 61.8% (95% CI = 58.8–64.7, *I*^2^ = 94.7). The prevalence of sleep disturbance decreased from 66.1% (95% CI = 46.7–81.3, *I*^2^ = 98.6) in studies published from 1998 to 2005 to 58.6% (95% CI = 48–68.4, *I*^2^ = 94.5) in studies published in 2021. In the subgroup analyses by continent, according to where the study was conducted, the pooled prevalence was 57.9% (95% CI = 45.4–69.4, *I*^2^ = 93.9), 60.5% (95% CI = 56.8–64, *I*^2^ = 97), 61% (95% CI = 56.5–65.4, *I*^2^ = 91.8), 62.7% (95% CI = 56.2–68.7, *I*^2^ = 96.3) for Australia, North America, Europe, and Asia, respectively.

In the subgroup analyses using the NOS, the pooled prevalence in studies with moderate risk of bias 60.9% (95% CI = 57.7–64.1, *I*^2^ = 96), whereas low risk of bias studies accounted for 60.9% (95% CI = 55.9–65.4, *I*^2^ = 97.6). Pooled prevalence of sleep disturbance was 58.7% (95% CI = 55.7–61.7, *I*^2^ = 97.6) in cross-sectional design studies and 63.5% (95% CI = 59–67.8, *I*^2^ = 97) in longitudinal design studies.

The pooled prevalence of sleep disturbance ranged from 44.8% (95% CI = 31.6–58.9, *I*^2^ = 92.6) in patients with prostate cancer to 64.4% (95% CI = 59.5–69.9, *I*^2^ = 97.7) in studies that included patients with mixed type of cancer. The pooled prevalence rates with regard to sleep disturbance were 57.7% (95% CI = 53.4–61.9, *I*^2^ = 96.2) in localized, and 70.8% (95% CI = 61.7–78.5, *I*^2^ = 96.5) in those studies that included advanced cancer patients’ stages. The treatment status had the highest prevalence of sleep disturbance in studies reporting mixed treatment status with a pooled prevalence of 63.8% (95% CI = 58.1–69.2, *I*^2^ = 96.7); this was followed by studies included patient on anti-cancer treatment studies with 60.2% (95% CI = 56.8–63.4, *I*^2^ = 93.9).

Regarding the scale used, the highest prevalence of sleep disturbance was found among studies that used the PSQI at 64% (95% CI = 61.4–66.5, *I*^2^ = 93.1), followed by studies using the GSDS at 61.5% (95% CI = 52.9–69.5, *I*^2^ = 94.6); whereas the lowest prevalence of sleep disturbance studies that used the ESS was 26.9% (95% CI = 20.7–34.2, *I*^2^ = 87) (Table 1).

**Table 1.** Prevalence of Sleep Disturbance by Subgroups Categories.

Subgroups	Categories	No. of studies	Sample size	Events	Prevalence (%)	95% CI (%)	*I*^2^ (%)	*p*
Age group								
	40–49	16	1,174	1926	67	65.3–76.2	93.6	<.001
	50–59	79	22,823	13,274	61.8	58.8–64.7	94.7	<.001
	60–69	49	8,440	16,324	58.6	53.2–63.8	97.4	<.001
	70–79	7	3,253	4,623	48.3	40–56.7	80.8	<.001
Year of publication								
	1998–2005	8	2,167	3,107	66.1	46.7–81.3	98.6	<.001
	2006–2010	30	4,298	8,383	60.6	54.7–66.1	95.5	<.001
	2011–2015	57	8,432	15,548	60.1	55.6–64.4	96.2	<.001
	2016–2020	57	10,429	17,223	60.8	56.7–64.8	95.9	<.001
	2021–	8	1,985	1,155	58.6	48–68.4	94.5	<.001
Continent								
	Australia	4	1,457	749	57.9	45.4–69.4	93.9	<.001
	North America	87	15,831	27,589	60.5	56.8–64	97	<.001
	Europe	22	3,676	7,196	61	56.5–65.4	91.8	<.001
	Asia	42	6,261	10,091	62.7	56.2–68.7	96.3	<.001
NOS								
	Low	30	3,875	6,899	60.9	55.9–65.7	97.6	<.001
	Moderate	130	27,638	15,854	60.9	57.7–64.1	96	<.001
Study design								
	CS	96	27,700	14,802	58.7	55.7–61.7	95.4	<.001
	LG	64	9,350	17,062	63.5	59–67.8	97	<.001
Type of cancer								
	Prostate	6	1891	3,499	44.8	31.6–58.9	92.6	<.001
	Head and neck	8	1,296	661	54.4	44.8–63.6	87.9	<.001
	Gastrointestinal	4	424	875	50.6	39.7–61.9	91.0	<.001
	Breast	64	19,542	10,489	59.7	56.2–63.2	95.2	<.001
	Lung	8	743	1,410	63.3	54.9–71.9	84.9	<.001
	Gynecological	7	748	1,118	63.6	51.8–73.9	93.7	<.001
	Mixed	57	9,279	17,111	64.4	59.5–69.9	97.7	<.001
Cancer stages								
	Localized	53	9,002	14,929	57.7	53.4–61.9	96.2	<.001
	Mixed	71	21,959	12,512	58.9	55.4–62.3	95.8	<.001
	No evidence of disease	16	3,782	6,194	67.9	54–79.2	97.5	<.001
	Advanced	20	2,886	6,821	70.8	61.7–78.5	96.5	<.001
Treatment								
	>3 months after/treat	52	8,945	16,171	59.7	54.6–64.6	97.4	<.001
	Under/treat	80	13,736	23,676	60.2	56.8–63.4	93.9	<.001
	Mixed	28	11,380	7,188	63.8	58.1–69.2	96.7	<.001
Instrument								
	ESS	8	3,997	8,350	26.9	20.7–34.2	87	<.001
	AIS	5	660	1,196	52.5	40–64.7	93.3	<.001
	ISI	31	4,231	7,438	59.9	53.3–66.2	97.3	<.001
	GSDS	11	943	1,452	61.5	52.9–69.5	94.6	<.001
	PSQI	96	23,348	14,255	64.0	61.4–66.5	93.1	<.001

CS: cross-sectional; LG: longitudinal; AIS: Athens insomnia scale; ESS: Epworth sleepiness scale; GSDS: general sleep disturbance scale; ISI: insomnia severity index; PSQI: Pittsburgh Sleep Quality Index.

Meta-regression analysis was conducted to evaluate the potential heterogeneity. The results showed that the female percentage (*B* = −.003, *z* = −2.14, *p* = .032) was significantly associated with a higher prevalence of sleep disturbance; however, the mean age (*B* = −.0123, *z* = −1.68, *p* = .092) was not associated with sleep disturbance prevalence.

### Publication Bias

**Figure 3.**
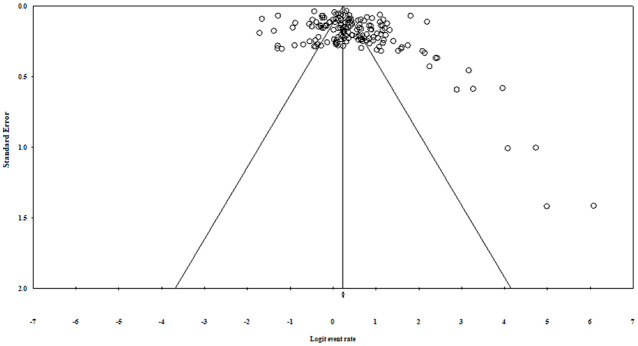
Funnel plot for assessing publication bias.

**Figure 4.**
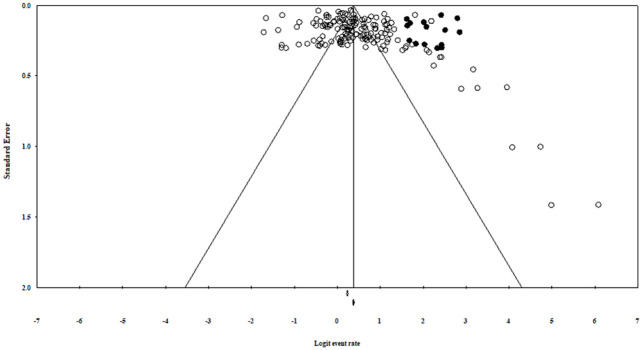
Adjusted funnel plot for assessing publication bias after adding 15 studies form the “trim and fill” analysis.

Funnel plots was asymmetrical (Figure 3) and Egger’s regression test (intercept = 2.33, 95% CI: 0.82–3.82, *z* = 3.07, *df* = 158, *p* = .001) which indicated presence of publication bias. However, further, Trim and Fill method was used, which identified 15 studies to be added (Figure 4) that did not show a significant impact on the pooled estimated prevalence of sleep disturbance.

## Discussion

Assessing the prevalence of sleep disturbance among patients with cancer is imperative for understanding the symptoms and necessary to identify the appropriate management strategy. This meta-analysis is the first to estimate the aggregate prevalence of sleep disturbance in patients with cancer. The aggregate prevalence of sleep disturbance in patients with cancer was 60.7%, which suggests that at least more than half of the cancer patients experience sleep disturbance. Those results are even higher when compared with sleep disturbance prevalence in other diseases such as cardiovascular (36.5%) (Edmealem et al., 2020), (37%) irritable bowel syndrome (Wang et al., 2018), diabetes (47%) (Birhanu et al., 2020), and cystic fibrosis (43%) (Mulette et al., 2021). Moreover, Simonelli et al. (2018), who conducted meta-analyses including 45 studies of low and middle-income countries from the general population, estimated the prevalence of sleep disturbance to be 32%. Another meta-analysis of 12 studies of the general population reported a prevalence of sleep disturbance. This highlights the importance of this meta-analysis to estimate the prevalence of sleep disturbance and suggests that the sleep disturbance was attributable to diseases related factors.

This meta-analysis found that the prevalence of sleep disturbance ranged from 15.3% (Akechi et al., 2007) and 99.8% (Mercadante et al., 2017) between studies. This variation of the prevalence between the studies could be attributed to the diversity of the assessment scales (cut-off of scales) or unique features of certain types of cancer and cancer treatment strategies that increase the probability of experiencing sleep disturbance. For example, as shown in Table 1, the cut-off score of PSQI in (Fong & Ho, 2020) was ≥8, whereas (Chan et al., 2021) used >5. The ISI cut off score was ≥7 in (Schieber et al., 2019) and ≥15 by (Mao et al., 2018). In ESS (Valko et al., 2015) used ≥10, while (Gibbins et al., 2009) used ≥15.

With respect to gender, the higher prevalence was reported by studies with a higher proportion of females than those with a lower proportion of female participants. Interestingly, 13 studies included in this meta-analysis show that female gender has a significant association with a high prevalence of sleep disturbance (Chung et al., 2017; Echchikhi et al., 2017; Ji et al., 2017; Johansen et al., 2018; Jung et al., 2016; Li, Otomaru et al., 2017; Saini et al., 2013; Santoso et al., 2021; Savard et al., 2009; Schieber et al., 2019; Strollo et al., 2020; Sun et al., 2020; Tejada et al., 2019). In addition, a meta-analysis involved 12 studies of the general population found that females were a significantly higher prevalence of sleep disturbance than the male prevalence of sleep disturbance (Zeng et al., 2020). This may be because, first, women are less likely to receive social support (Drageset et al., 2016), second women are more likely to suffer from mental illnesses such as depression and anxiety (Zhao et al., 2020) which may increase sleep disturbance.

There was no association between mean age and overall sleep disturbance prevalence rate. Thirty-nine studies involved in this pooled analysis found a significant association between age and sleep disturbance prevalence (Al Maqbali et al., 2021; Bagheri-Nesami et al., 2016; Bardwell et al., 2008; Barsevick et al., 2010; Beck et al., 2010; Berger et al., 2019a; Caplette-Gingras et al., 2013; Chung et al., 2017; Colagiuri et al., 2011; Davies et al., 2017; Desai et al., 2013; Dirksen et al., 2009; Echchikhi et al., 2017; Fekih-Romdhane et al., 2020; Fleming et al., 2019; Grutsch et al., 2011; Halle et al., 2017; Klyushnenkova et al., 2015; Liou et al., 2019; Li et al., 2017a; Liu et al., 2012; Mansano-Schlosser & Ceolim, 2012; Mercadante et al., 2015; Miaskowski et al., 2011; Morris et al., 2015; Palesh et al., 2007, 2008, 2010; Rogers et al., 2008; Santoso et al., 2021; Savard et al., 2005a; Steel et al., 2018; Strollo et al., 2020; Sun et al., 2020; Taylor et al., 2012; Tejada et al., 2019; Yoshikawa et al., 2020; Zubair et al., 2019). This was because there are differences in sleep disturbance prevalence depending on the stage of treatment. Based on the data of 129 studies, sleep disturbance prevalence was 59.7% among those with more than 3 months after curative treatment completed; 60.2% during anti-cancer treatment; and 64.2% in mixed stage of treatment studies. This could be explained because there were 15 mixed treatment studies (Chung et al., 2017; Davies et al., 2017; Echchikhi et al., 2017; Kotronoulas et al., 2011; Lin et al., 2020; Mansano-Schlosser & Ceolim, 2012; Mercadante et al., 2015, 2017; Morris et al., 2015; Nakamura et al., 2013; Phillips et al., 2012; Savard et al., 2009; Savard et al., 2005b; Tzeng et al., 2012; Vargas et al., 2010) that reported on sleep disturbance in the mixed type of cancer studies, which reported to experience the greatest sleep disturbance among the cancer type in this meta-analyses. The percentage of sleep disturbance during anti-cancer treatments was expected, as sleep disturbance typically increases during radiation therapy (Van Onselen et al., 2010), chemotherapy (Yoshikawa et al., 2020), and biological therapy (Gonzalez et al., 2018). In addition, treatment introduces various toxicities to patients, which will likely increase the experience of sleep disturbance (Pillai et al., 2018; Song & Bai, 2021).

A high prevalence of sleep disturbance occurred in studies with a mixed type of cancer (64.4%), specifically gynecological (63.6%) and lung (63.3%). These results could be explained as this meta-analysis included seven gynecological types of cancer, which six of them (Clevenger et al., 2012, 2013; Li et al., 2017a; Sandadi et al., 2011; Tian et al., 2015; Yu & Nho, 2015) reported patients during anti-cancer treatments, and eight lung types of cancer of which five (Chen et al., 2008; Dreher et al., 2018; Grutsch et al., 2011; Gu et al., 2018; Halle et al., 2017) reported patients during anti-cancer treatments.

Further, the result of this meta-analysis also suggests that the studies involving advanced cancer patients were highest in reporting sleep disturbance with 70.8%, followed by studies that included no evidence of disease of cancer at 67.9%. The result of these meta-analyses found differences in the prevalence of sleep disturbance according to different cancer type subgroups. A patient diagnosed with breast (59.7%) and gastrointestinal (50.6%) cancers reported the highest sleep disturbance compared with patients with gastrointestinal (50.6%) and prostate (44.8%). This may be attributable to unequal features of the sample, the side effect treatment phases, and time point of measurement (Ancoli-Israel, 2015). Unfortunately, this review was unable to assess the influences factors of sleep disturbance prevalence rate. In addition, in this review, nine scales have been employed to assess sleep disturbance in 159 studies. The Pittsburgh Sleep Quality Index (PSQI) had the highest prevalence of sleep disturbance (64%), whereas Epworth sleepiness scale (ESS) showed the lowest prevalence of sleep disturbance at 26.9%, according to the scales used. The discrepancy may be due to the differences in studies participant characteristics.

The current review used different search terms and databases than did the previous reviews (Leysen et al., 2019; Santoso et al., 2019), and the inclusion criteria were different. In addition, this review included more studies because it used different MeSH (Medical Subject Headings) terms and databases. The review investigates the prevalence of sleep disturbance in differences type of patients with cancer, whereas the other reviews related to specific types of cancer. An important point is that this review pooled the prevalence of sleep disturbance from a very large sample (*N* = 46,279), and participants were recruited from several countries. Consequently, generalization of the findings is more likely to be strong.

The findings of this review have several practical and research implications. First, it would be advisable for healthcare professionals to conduct regular assessments with regard to sleep disturbance in clinical settings in order to help patients with cancer detect and overcome sleep disturbance. Second, government and healthcare providers need to identify or design appropriate guidelines to assist healthcare professionals to make appropriate decisions for cancer patients. There are several international clinical guidelines with regard to sleep disturbance, developed by different organizations, that can help improve the sleep of cancer patients (Berger et al., 2019b; National Comprehensive Cancer Network, 2021; National Institute for Health and Care Excellence, 2021; Sateia et al., 2017; Schutte-Rodin et al., 2008). These guidelines provide strategies for managing sleep disturbance in terms of clinical practice, such as the screening, assessment and management of sleep disturbance for healthcare professionals (Howell et al., 2014). The most common form of evidence-based treatment for sleep disturbance is cognitive behavior therapy (Ma et al., 2021), exercise (Tang et al., 2019) and Mindfulness-based stress reduction(Suh et al., 2021). Furthermore, Choi et al. (2018) suggested the need for self-help management applications, and training healthcare professionals to detect and treat sleep disturbance for different populations.

This meta-analysis was performed with strict inclusion criteria to reduce heterogeneity. Additionally, the studies’ quality was assessed using the NOS; all studies fell into medium-quality and low-quality categories. The bias mainly involved the selection and size of the sample and follow-up time. Therefore, the amount of heterogeneity between the studies in pooled prevalence and subgroup analyses was low. Besides, sensitivity analysis by excluding one study at a time and trim-and-fill method were shown the robustness of this meta-analysis.

The major strength of this meta-analysis is the large sample size of over 46,279 subjects drawn from 160 studies estimated sleep disturbance in patients with cancer. However, there are several potential limitations in this meta-analysis. First, there is a possibility that some studies were not included in this meta-analysis, although this analysis used different MeSH terms and several databases. In addition, only studies published in English were included in this analysis. Second, sleep disturbance was assessed using various scales measures; this led to variability between studies and could increase the errors of prevalence estimates. Third, there were insufficient data available on the demographic and clinical characteristics, so not all information could be eliminated thoroughly. Fourth, sleep disturbance is a very complex and multidimensional condition such as difficulty falling asleep, problems with the initiation and maintenance of sleep, poor sleep timing, quality, efficiency, and excessive daytime sleepiness (Al Maqbali, 2020; Strik et al., 2021). For this reason, this meta-analysis only reported the total scores of the questionnaires, no specific dimensions of sleep disturbance were assessed. Consequently, future systematic review and meta-analysis need to identify specific prevalence of subcategory of sleep disturbance and the associated risk factors. Finally, the funnel plot and Eggers’s test may suggest existing publication bias; however, the pooled prevalence of sleep disturbance remained stable after using Trim-and-fill method.

## Conclusion

This is the first systematic review and meta-analysis reporting pooled prevalence estimates for sleep disturbance among patients with cancer. The findings show that over half of patients with cancer have experience of sleep disturbance, which is higher than the general population and other diseases. These results highlight the need for clear management strategies that can reduce sleep disturbance among cancer patients. Furthermore, more work should be done to reduce sleep disturbance through assessment and management of sleep status in patients with cancer.

## Supplemental Material

sj-docx-1-cnr-10.1177_10547738221092146 – Supplemental material for Prevalence of Sleep Disturbance in Patients With Cancer: A Systematic Review and Meta-Analysis Click here for additional data file. Supplemental material, sj-docx-1-cnr-10.1177_10547738221092146 for Prevalence of Sleep Disturbance in Patients With Cancer: A Systematic Review and Meta-Analysis by Mohammed Al Maqbali, Mohammed Al Sinani, Ahmad Alsayed and Alexander M. Gleason in Clinical Nursing Research

sj-docx-2-cnr-10.1177_10547738221092146 – Supplemental material for Prevalence of Sleep Disturbance in Patients With Cancer: A Systematic Review and Meta-Analysis Click here for additional data file. Supplemental material, sj-docx-2-cnr-10.1177_10547738221092146 for Prevalence of Sleep Disturbance in Patients With Cancer: A Systematic Review and Meta-Analysis by Mohammed Al Maqbali, Mohammed Al Sinani, Ahmad Alsayed and Alexander M. Gleason in Clinical Nursing Research
