# Supplementary material for: Prevalence of Sleep Disturbance in Patients With Cancer: A Systematic Review and Meta-Analysis
Source: Clin Nurs Res. 2022 Apr 28;31(6):1107–23. doi: 10.1177/10547738221092146 (PMC9266067; doi:10.1177/10547738221092146)
Supplement: sj-docx-1-cnr-10.1177_10547738221092146 – Supplemental material for Prevalence of Sleep Disturbance in Patients With Cancer: A Systematic Review and Meta-Analysis [file sj-docx-1-cnr-10.1177_10547738221092146.docx]

**Table 1:** Characteristics of the Included 160 Studies

|  | Study | Events | N | Female | Age Mean | Age Group | Year of Publication | Country | Continent | Study Design | Type of Cancer | Cancer Stage | Treatment Status | Instrument | Cut Off | NOS |
| --- | --- | --- | --- | --- | --- | --- | --- | --- | --- | --- | --- | --- | --- | --- | --- | --- |
| 1 | (Carpenter and Andrykowski, 1998a) [1] | 68 | 102 | 102 | 56.5 |  | 1998 | US | North America | CS | Breast | Localized | Mixed | PSQI | ˃8 | Moderate |
| 2 | (Savard et al., 2001) [2] | 145 | 300 | 300 | 60 | 28-90 | 2001 | Canada | North America | CS | Breast | Localized | Under/Treat | SII | NG | Moderate |
| 3 | (Fortner et al., 2002) [3] | 48 | 72 | 72 | 51.3 |  | 2002 | US | North America | CS | Breast | Localized | Mixed | PSQI | ˃5 | Moderate |
| 4 | (Beck et al., 2004) [4] | 245 | 473 | 334 | 56.6 | 26-83 | 2004 | US | North America | LG | Mixed | Mixed | > 3 month After/Treat | PSQI | ˃8 | Moderate |
| 5 | (Savard et al., 2005b) [5] | 1436 | 1670 | 785 |  | 40-76 | 2005 | Canada | North America | LG | Mixed | Localized | Mixed | ISI | ≥15 | Moderate |
| 6 | (Savard et al., 2005a) [6] | 96 | 327 | 0 | 66 | 47-80 | 2005 | Canada | North America | LG | Prostate | Localized | Mixed | ISI | ≥8 | Moderate |
| 7 | (Sela et al., 2005) [7] | 72 | 100 | 48 | 60.1 | 21-68 | 2005 | Canada | North America | LG | Mixed | Advanced | > 3 month After/Treat | SQ | ≥5 | Moderate |
| 8 | (Carlson and Garland, 2005) [8] | 57 | 63 | 49 | 54 | 32-78 | 2005 | Canada | North America | LG | Mixed | Mixed | Under/Treat | PSQI | ˃5 | Moderate |
| 9 | (Ancoli-Israel et al., 2006) [9] | 53 | 77 | 75 | 51 | 34-79 | 2006 | US | North America | CS | Breast | Localized | Under/Treat | PSQI | ˃5 | Moderate |
| 10 | (Gooneratne et al., 2007) [10] | 43 | 76 | 47 | 73.6 | 61-89 | 2007 | US | North America | CS | Lung | No Evidence of Disease | > 3 month After/Treat | PSQI | ˃5 | Moderate |
| 11 | (Berger et al., 2007) [11] | 72 | 127 | 127 | 51.4 | 34-83 | 2007 | US | North America | CS | Breast | Localized | Mixed | PSQI | ˃5 | Low |
| 12 | (Fox et al., 2007) [12] | 73 | 73 | 34 | 46 | 22-92 | 2007 | US | North America | CS | Brain | Localized | Under/Treat | GSDS | ≥ 43 | Moderate |
| 13 | (Palesh et al., 2007) [13] | 60 | 93 | 93 | 53.8 | 33-80 | 2007 | US | North America | CS | Breast | Advanced | > 3 month After/Treat | SQ | ≥5 | Moderate |
| 14 | (Akechi et al., 2007) [14] | 32 | 209 | 72 | 59.6 |  | 2007 | Japan | Asia | LG | Mixed | Advanced | > 3 month After/Treat | Clinical Int | NG | Low |
| 15 | (Mystakidou et al., 2007) [15] | 82 | 102 | 56 | 62.8 | 26-87 | 2007 | Greek | Europe | CS | Mixed | Advanced | > 3 month After/Treat | PSQI | ˃8 | Moderate |
| 16 | (Shahidi et al., 2007) [16] | 39 | 53 | 22 | 47 | 18-78 | 2007 | Iran | Asia | LG | Breast | Mixed | Mixed | PSQI | ˃5 | Moderate |
| 17 | (Bardwell et al., 2008) [17] | 1039 | 2645 | 2654 | 53 | 28-74 | 2008 | US | North America | CS | Breast | Localized | > 3 month After/Treat | WHI-SDS | ≥ 9 | Low |
| 18 | (Palesh et al., 2008) [18] | 91 | 99 | 99 | 54.6 | 36-80 | 2008 | US | North America | CS | Breast | Advanced | > 3 month After/Treat | Actigraphy |  | Low |
| 19 | (Rogers et al., 2008) [19] | 30 | 58 | 43 | 60 |  | 2008 | US | North America | CS | Head and Neck | Mixed | Mixed | PSQI | ˃5 | Moderate |
| 20 | (Chen et al., 2008) [20] | 79 | 115 | 41 | 59.4 |  | 2008 | Taiwan | Asia | LG | Lung | Mixed | Under/Treat | PSQI | ˃5 | Moderate |
| 21 | (Dirksen et al., 2009a) [21] | 48 | 86 | 86 | 57.8 | 29-86 | 2009 | US | North America | CS | Breast | No Evidence of Disease | > 3 month After/Treat | ISI | ≥15 | Moderate |
| 22 | (Tremblay et al., 2009) [22] | 30 | 57 | 57 | 54 |  | 2009 | Canada | North America | CS | Breast | No Evidence of Disease | > 3 month After/Treat | ISI | ≥8 | Moderate |
| 23 | (Liu et al., 2009) [23] | 50 | 76 | 76 | 51.1 |  | 2009 | US | North America | CS | Breast | Localized | Under/Treat | PSQI | ˃5 | Low |
| 24 | (Dirksen et al., 2009b) [24] | 27 | 51 | 0 | 74 | 52-94 | 2009 | US | North America | CS | Prostate | Mixed | Mixed | ISI | ≥8 | Moderate |
| 25 | (Gibbins et al., 2009) [25] | 28 | 60 | 33 | 67 | 32-80 | 2009 | US | North America | CS | Mixed | Advanced | > 3 month After/Treat | ESS | ≥15 | Moderate |
| 26 | (Mystakidou et al., 2009) [26] | 79 | 82 | 46 | 62.6 | 26-87 | 2009 | Greek | Europe | CS | Mixed | Advanced | > 3 month After/Treat | PSQI | ˃5 | Moderate |
| 27 | (Aouizerat et al., 2009) [27] | 75 | 168 | 75 | 60.9 |  | 2009 | US | North America | LG | Mixed | Mixed | Under/Treat | GSDS | ≥ 43 | Low |
| 28 | (Price et al., 2009) [28] | 348 | 796 | 796 | 60.5 | 22-82 | 2009 | Australia | Australia | LG | Gynaecological | Mixed | Mixed | ISI | ≥7 | Low |
| 29 | (Savard et al., 2009a) [29] | 559 | 939 | 630 | 57.1 |  | 2009 | Canada | North America | LG | Mixed | Mixed | Mixed | Clinical Int | NG | Low |
| 30 | (Berger et al., 2009) [30] | 58 | 206 | 206 | 51 | 29-83 | 2009 | US | North America | LG | Breast | Localized | Under/Treat | PSQI | ≥8 | Moderate |
| 31 | (Palesh et al., 2010) [31] | 362 | 823 | 593 | 58 | 22-93 | 2010 | US | North America | LG | Mixed | Mixed | Under/Treat | HDI | NG | Moderate |
| 32 | (Van Onselen et al., 2010) [32] | 102 | 178 | 85 | 60.1 |  | 2010 | US | North America | CS | Mixed | Mixed | Under/Treat | PSQI | ˃5 | Moderate |
| 33 | (Vargas et al., 2010) [33] | 170 | 240 | 240 | 50.2 |  | 2010 | US | North America | CS | Mixed | Mixed | Mixed | PSQI | ˃5 | Moderate |
| 34 | (Otte et al., 2010a) [34] | 160 | 246 | 246 | 48.2 |  | 2010 | US | North America | CS | Breast | No Evidence of Disease | > 3 month After/Treat | PSQI | ˃5 | Moderate |
| 35 | (Dodd et al., 2010a) [35] | 63 | 112 | 112 | 50.1 | 28-78 | 2010 | US | North America | LG | Breast | Localized | Under/Treat | GSDS | ≥ 43 | Moderate |
| 36 | (Barsevick et al., 2010) [36] | 222 | 292 | 240 | 53.9 |  | 2010 | US | North America | LG | Mixed | Mixed | Under/Treat | PSQI | ˃5 | Moderate |
| 37 | (Beck et al., 2010) [37] | 119 | 183 | 183 | 50.7 | 28-75 | 2010 | US | North America | LG | Breast | Localized | Under/Treat | PSQI | ˃5 | Moderate |
| 38 | (Park et al., 2010) [38] | 72 | 94 | 94 | 52 |  | 2010 | Korea | Asia | CS | Breast | Localized | Under/Treat | PSQI | ˃5 | Moderate |
| 39 | (Garrett et al., 2011) [39] | 85 | 160 | 78 | 60.9 |  | 2011 | US | North America | CS | Mixed | Mixed | Under/Treat | PSQI | ˃5 | Moderate |
| 40 | (Miaskowski et al., 2011b) [40] | 59 | 82 | 00 | 67.1 |  | 2011 | US | North America | LG | Prostate | Localized | Mixed | GSDS | ≥ 43 | Low |
| 41 | (Miaskowski et al., 2011a) [41] | 77 | 185 | 89 | 60.56 | 24-85 | 2011 | US | North America | LG | Mixed | Localized | Under/Treat | GSDS | ≥ 43 | Moderate |
| 42 | (Sandadi et al., 2011) [42] | 58 | 86 | 86 | 58.1 | 19-89 | 2011 | US | North America | CS | Gynaecological | Mixed | Under/Treat | PSQI | ≥5 | Moderate |
| 43 | (Delgado-Guay et al., 2011) [43] | 86 | 101 | 53 | 60 | 25-84 | 2011 | US | North America | CS | Mixed | Advanced | Under/Treat | PSQI | ˃5 | Moderate |
| 44 | (Bower et al., 2011) [44] | 67 | 103 | 103 | 51.2 | 32-66 | 2011 | US | North America | LG | Breast | Localized | Under/Treat | PSQI | ˃5 | Moderate |
| 45 | (Hanisch et al., 2011) [45] | 14 | 60 | 0 | 71.4 | 54-88 | 2011 | US | North America | CS | Prostate | Localized | Under/Treat | ESS | ≥10 | Moderate |
| 46 | (Colagiuri et al., 2011) [46] | 1738 | 3002 | 3002 | 54.4 | 26-70 | 2011 | Denmark | Europe | CS | Breast | Mixed | Mixed | PSQI | ˃5 | Low |
| 47 | (Moore et al., 2011) [47] | 124 | 206 | 206 | 52.13 | 29-79 | 2011 | US | North America | LG | Breast | Localized | Under/Treat | PSQI | ˃5 | Moderate |
| 48 | (Grutsch et al., 2011) [48] | 70 | 84 | 19 | 62 | 40-94 | 2011 | US | North America | LG | Lung | Advanced | Under/Treat | PSQI | ˃5 | Moderate |
| 49 | (Sun et al., 2011) [49] | 84 | 195 | 116 | 56.7 |  | 2011 | Taiwan | Asia | CS | Mixed | Mixed | > 3 month After/Treat | AIS | ˃7 | Moderate |
| 50 | (Rand et al., 2011) [50] | 239 | 395 | 395 | 59.3 | 35-85 | 2011 | Norway | Europe | CS | Mixed | Mixed | Under/Treat | PSQI | ˃5 | Moderate |
| 51 | (Kotronoulas et al., 2011) [51] | 114 | 209 | 124 | 54.9 | 19-85 | 2011 | Greek | Europe | CS | Mixed | Mixed | Mixed | PSQI | ≥8 | Moderate |
| 52 | (Enderlin et al., 2011) [52] | 47 | 67 | 67 | 65.1 | 50-90 | 2011 | US | North America | CS | Breast | Mixed | Mixed | PSQI | ˃5 | Moderate |
| 53 | (Dhruva et al., 2012) [53] | 40 | 73 | 73 | 55 |  | 2012 | US | North America | LG | Breast | Mixed | Under/Treat | GSDS | ≥ 43 | Moderate |
| 54 | (Phillips et al., 2012) [54] | 165 | 288 | 187 | 56.7 |  | 2012 | US | North America | CS | Mixed | Mixed | Mixed | PSQI | ˃5 | Moderate |
| 55 | (Forsythe et al., 2012) [55] | 255 | 1171 | 910 | 64.3 |  | 2012 | US | North America | CS | Mixed | Mixed | > 3 month After/Treat | ESS | ≥10 | Moderate |
| 56 | (Liu et al., 2012b) [56] | 59 | 97 | 97 | 50.7 | 34-79 | 2012 | US | North America | LG | Breast | Localized | Under/Treat | PSQI | ˃5 | Low |
| 57 | (Mosher and Duhamel, 2012) [57] | 63 | 90 | 90 | 57.9 |  | 2012 | US | North America | CS | Breast | Advanced | > 3 month After/Treat | PSQI | ˃5 | Low |
| 58 | (Liu et al., 2012a) [58] | 40 | 53 | 53 | 50.3 | 34-79 | 2012 | US | North America | LG | Breast | Localized | Under/Treat | PSQI | ˃5 | Moderate |
| 59 | (Clevenger et al., 2012) [59] | 103 | 133 | 133 | 60.4 |  | 2012 | US | North America | LG | Gynaecological | Mixed | Under/Treat | PSQI | ˃5 | Moderate |
| 60 | (Van Onselen et al., 2012) [60] | 219 | 398 | 398 | 54.9 |  | 2012 | US | North America | LG | Breast | Localized | Under/Treat | GSDS | ≥ 43 | Low |
| 61 | (Mansano-Schlosser and Ceolim, 2012) [61] | 78 | 140 | 72 | 69.8 |  | 2012 | Brazil | South America | CS | Mixed | Mixed | Mixed | PSQI | ˃5 | Moderate |
| 62 | (Taylor et al., 2012) [62] | 22 | 51 | 51 | 64 | 31-87 | 2012 | US | North America | CS | Breast | Localized | > 3 month After/Treat | ISI | ≥7 | Moderate |
| 63 | (Tzeng et al., 2012) [63] | 162 | 205 | 70 | 58.4 |  | 2012 | Taiwan | Asia | CS | Mixed | Mixed | Mixed | PSQI | ˃5 | Moderate |
| 64 | (Desai et al., 2013) [64] | 207 | 413 | 413 | 61.7 | 33-88 | 2013 | US | North America | CS | Breast | Localized | Under/Treat | ISI | ≥8 | Moderate |
| 65 | (Clevenger et al., 2013) [65] | 123 | 173 | 173 | 59.4 |  | 2013 | US | North America | LG | Gynaecological | Mixed | Under/Treat | PSQI | ˃5 | Moderate |
| 66 | (Sanford et al., 2013) [66] | 53 | 80 | 80 | 49.7 | 29-71 | 2013 | US | North America | LG | Breast | Localized | Under/Treat | PSQI | ≥5 | Moderate |
| 67 | (Saini et al., 2013) [67] | 102 | 173 | 79 | 58.8 | 20-80 | 2013 | Italy | Europe | CS | Mixed | Mixed | Under/Treat | PSQI | ≥5 | Moderate |
| 68 | (Caplette-Gingras et al., 2013) [68] | 47 | 63 | 63 | 50 |  | 2013 | Canada | North America | CS | Breast | Localized | Under/Treat | ISI | ≥7 | Moderate |
| 69 | (Nakamura et al., 2013) [69] | 30 | 57 | 43 | 52 |  | 2013 | US | North America | LG | Mixed | Mixed | Mixed | MOS-SS | 35 | Moderate |
| 70 | (Hall et al., 2014) [70] | 187 | 313 | 313 | 43.9 | 24-50 | 2014 | US | North America | CS | Breast | No Evidence of Disease | > 3 month After/Treat | ISI | ≥8 | Moderate |
| 71 | (Doong et al., 2015) [71] | 230 | 393 | 393 | 54.2 |  | 2014 | US | North America | CS | Breast | Mixed | Under/Treat | GSDS | ≥ 43 | Moderate |
| 72 | (Ho and Fong, 2014) [72] | 91 | 197 | 197 | 49.4 |  | 2014 | Hong Kong | Asia | LG | Breast | Localized | Under/Treat | PSQI | ≥8 | Low |
| 73 | (Ma et al., 2014) [73] | 25 | 68 | 34 | 52.4 |  | 2014 | Taiwan | Asia | CS | Mixed | Mixed | > 3 month After/Treat | PSQI | ≥5 | Moderate |
| 74 | (Hong et al., 2014) [74] | 486 | 706 | 257 | 52.4 |  | 2014 | China | Asia | CS | Mixed | Mixed | Under/Treat | PSQI | ≥5 | Moderate |
| 75 | (Mao et al., 2014) [75] | 55 | 67 | 67 | 59.7 | 41-76 | 2014 | US | North America | LG | Breast | Localized | Under/Treat | PSQI | ˃5 | Moderate |
| 76 | (Romito et al., 2014) [76] | 302 | 403 | 252 | 57 | 20-86 | 2014 | Italy | Europe | CS | Mixed | Mixed | Under/Treat | PSQI | ≥5 | Low |
| 77 | (Vargas et al., 2014) [77] | 168 | 240 | 240 | 50 |  | 2014 | US | North America | LG | Breast | Localized | Under/Treat | PSQI | ˃5 | Low |
| 78 | (Courneya et al., 2014) [78] | 152 | 296 | 296 | 50.9 |  | 2014 | Canada | North America | LG | Breast | Localized | Under/Treat | PSQI | ˃5 | Low |
| 79 | (Kashani and Kashani, 2014) [79] | 54 | 57 | 57 | 43 | 23-63 | 2014 | Iran | Asia | LG | Breast | Localized | Under/Treat | PSQI | ˃5 | Moderate |
| 80 | (Valko et al., 2015) [80] | 14 | 65 | 21 | 57.3 |  | 2015 | Switzerland | Europe | CS | Brain | Localized | Under/Treat | ESS | ≥10 | Moderate |
| 81 | (Ho et al., 2015) [81] | 101 | 134 | 134 | 50.7 |  | 2015 | US | North America | LG | Breast | Localized | Under/Treat | PSQI | ˃5 | Moderate |
| 82 | (Klyushnenkova et al., 2015) [82] | 138 | 861 | 861 | 62 | 33-95 | 2015 | US | North America | CS | Breast | No Evidence of Disease | > 3 month After/Treat | ESS | ≥10 | Moderate |
| 83 | (Mercadante et al., 2015) [83] | 499 | 890 | 391 | 69.7 |  | 2015 | Italy | Europe | CS | Mixed | Advanced | Mixed | AIS | ≥6 | Low |
| 84 | (He et al., 2015) [84] | 88 | 162 | 124 | 40.2 |  | 2015 | China | Asia | CS | Head and Neck | Mixed | Under/Treat | PSQI | ˃5 | Moderate |
| 85 | (Nishiura et al., 2015) [85] | 28 | 50 | 15 | 71.8 |  | 2015 | Japan | Asia | CS | Lung | Advanced | Mixed | AIS | ≥6 | Moderate |
| 86 | (Yu and Nho, 2015) [86] | 97 | 152 | 152 | 54.1 | 22-72 | 2015 | Korea | Asia | CS | Gynaecological | Mixed | Under/Treat | PSQI | ≥5 | Moderate |
| 87 | (Qin et al., 2015) [87] | 31 | 60 | 23 | 39.3 |  | 2015 | China | Asia | CS | Head and Neck | Advanced | Under/Treat | PSQI | ≥5 | Moderate |
| 88 | (Morris et al., 2015) [88] | 297 | 500 | 380 |  | 20-80 | 2015 | Australia | Australia | CS | Mixed | Mixed | Mixed | ISI | ≥7 | Low |
| 89 | (Tian et al., 2015) [89] | 49 | 76 | 76 | 48.4 |  | 2015 | China | Asia | LG | Gynaecological | Localized | Under/Treat | PSQI | ˃5 | Moderate |
| 90 | (Savard et al., 2015) [90] | 305 | 465 | 465 | 54.9 |  | 2015 | Canada | North America | LG | Breast | Localized | Under/Treat | ISI | ˃8 | Low |
| 91 | (Savard et al., 2015) [90] | 96 | 263 | 0 | 61.7 |  | 2015 | Canada | North America | LG | Prostate | Localized | Under/Treat | ISI | ˃8 | Low |
| 92 | (Akman et al., 2015) [91] | 127 | 314 | 167 | 58.5 | 28-84 | 2015 | Turkey | Asia | CS | Mixed | Mixed | Under/Treat | PSQI | ˃5 | Moderate |
| 93 | (Berrett-Abebe et al., 2015) [92] | 35 | 67 | 41 | 51.8 |  | 2015 | US | North America | CS | Mixed | No Evidence of Disease | > 3 month After/Treat | PSQI | ˃5 | Moderate |
| 94 | (Roscoe et al., 2015) [93] | 88 | 96 | 84 | 56 |  | 2015 | US | North America | LG | Mixed | No Evidence of Disease | > 3 month After/Treat | ISI | ˃8 | Low |
| 95 | (Lafçi and Öztunç, 2015) [94] | 59 | 60 | 60 | 46.4 | 27-69 | 2015 | Turkey | Asia | LG | Breast | Mixed | Under/Treat | PSQI | ˃5 | Moderate |
| 96 | (Li et al., 2017a) [95] | 52 | 95 | 95 | 57.4 |  | 2016 | Taiwan | Asia | CS | Gynaecological | Mixed | Under/Treat | PSQI | ˃5 | Moderate |
| 97 | (Jung et al., 2016) [96] | 40 | 198 | 198 | 46.3 |  | 2016 | Korea | Asia | LG | Breast | Localized | Under/Treat | ISI | ≥8 | Moderate |
| 98 | (Collins et al., 2017) [97] | 172 | 292 | 105 | 62 |  | 2016 | US | North America | CS | Mixed | Advanced | > 3 month After/Treat | PSQI | ˃5 | Moderate |
| 99 | (Bagheri-Nesami et al., 2016) [98] | 89 | 100 | 53 | 44 |  | 2016 | Iran | Asia | CS | Lymphoma | Mixed | Under/Treat | PSQI | ˃5 | Moderate |
| 100 | (Fontes et al., 2017) [99] | 302 | 501 | 501 |  |  | 2017 | Portugal | Europe | LG | Breast | Mixed | > 3 month After/Treat | PSQI | ˃5 | Low |
| 101 | (Loth et al., 2017) [100] | 20 | 51 | 14 | 61.1 |  | 2017 | France | Europe | CS | Head and Neck | Advanced | Under/Treat | ESS | ≥10 | Moderate |
| 102 | (Mercadante et al., 2017) [101] | 219 | 219 | 108 | 65.4 | 23-90 | 2017 | Italy | Europe | CS | Mixed | Advanced | Mixed | AIS | ≥6 | Moderate |
| 103 | (Halle et al., 2017) [102] | 161 | 264 | 112 | 65.8 |  | 2017 | Norway | Europe | LG | Lung | Mixed | Under/Treat | GSDS | ≥ 43 | Moderate |
| 104 | (Chung et al., 2017) [103] | 119 | 124 | 77 | 55.1 | 25-84 | 2017 | Korea | Asia | CS | Mixed | Mixed | Mixed | ISI | ≥7 | Moderate |
| 105 | (Ji et al., 2017) [104] | 74 | 170 | 97 | 56.6 | 32-77 | 2017 | China | Asia | LG | Mixed | Mixed | Under/Treat | PSQI | ˃5 | Moderate |
| 106 | (Davies et al., 2017) [105] | 123 | 174 | 100 | 66 | 35-90 | 2017 | UK | Europe | CS | Mixed | Advanced | Mixed | PSQI | ≥5 | Moderate |
| 107 | (Echchikhi et al., 2017) [106] | 136 | 284 | 215 | 49.5 | 17-80 | 2017 | Morocco | Africa | CS | Mixed | Mixed | Mixed | ISI | ≥7 | Moderate |
| 108 | (Rogers et al., 2017) [107] | 171 | 222 | 222 | 54.4 |  | 2017 | US | North America | LG | Breast | No Evidence of Disease | > 3 month After/Treat | PSQI | ˃5 | Low |
| 109 | (Cha et al., 2017) [108] | 95 | 208 | 165 | 50.7 | 19-81 | 2017 | Korea | Asia | CS | Mixed | Mixed | > 3 month After/Treat | ISI | ˃15 | Moderate |
| 110 | (Peoples et al., 2017a) [109] | 85 | 95 | 84 | 56.2 | 26-75 | 2017 | US | North America | LG | Mixed | No Evidence of Disease | > 3 month After/Treat | ISI | ˃8 | Moderate |
| 111 | (Mansano-Schlosser et al., 2017) [110] | 90 | 156 | 156 |  |  | 2017 | Brazil | South America | CS | Breast | Localized | Under/Treat | PSQI | ˃5 | Moderate |
| 112 | (Li et al., 2017b) [111] | 64 | 77 | 25 | 67.7 | 26-86 | 2017 | Japan | Asia | CS | Head and Neck | No Evidence of Disease | > 3 month After/Treat | PSQI | ˃5 | Moderate |
| 113 | (Miladinia et al., 2018) [112] | 274 | 406 | 186 | 33.4 |  | 2018 | Iran | Asia | CS | Leukaemia | Mixed | Under/Treat | PSQI | ˃5 | Moderate |
| 114 | (Gu et al., 2018) [113] | 92 | 202 |  | 61.2 |  | 2018 | China | Asia | CS | Lung | Mixed | Under/Treat | PSQI | ˃5 | Moderate |
| 115 | (Adams et al., 2018) [114] | 18 | 62 | 0 | 43.7 | 21-61 | 2018 | Canada | North America | LG | Testicular | Localized | > 3 month After/Treat | PSQI | ˃5 | Moderate |
| 116 | (Steel et al., 2018a) [115] | 173 | 294 | 106 | 61.9 |  | 2018 | US | North America | CS | Gastrointestinal | Advanced | > 3 month After/Treat | PSQI | ˃5 | Moderate |
| 117 | (Henneghan et al., 2018) [116] | 57 | 90 | 90 | 49 |  | 2018 | US | North America | CS | Breast | Localized | > 3 month After/Treat | PSQI | ≥6 | Moderate |
| 118 | (Jim et al., 2018) [117] | 257 | 570 | 241 | 54.8 |  | 2018 | US | North America | LG | Mixed | Mixed | > 3 month After/Treat | PSQI | ≥5 | Moderate |
| 119 | (Nelson et al., 2018) [118] | 35 | 84 | 60 | 38 |  | 2018 | US | North America | CS | Mixed | Mixed | > 3 month After/Treat | ISI | ≥8 | Moderate |
| 120 | (Gonzalez et al., 2018) [119] | 46 | 78 | 0 | 68.5 |  | 2018 | US | North America | CS | Prostate | Localized | > 3 month After/Treat | ISI | ≥8 | Moderate |
| 121 | (Dreher et al., 2018) [120] | 74 | 100 | 33 | 68.1 |  | 2018 | Germany | Europe | LG | Lung | Mixed | Under/Treat | PSQI | ≥5 | Moderate |
| 122 | (Mao et al., 2018) [121] | 605 | 1103 | 1103 | 63.2 | 20-92 | 2018 | US | North America | LG | Breast | Localized | > 3 month After/Treat | ISI | ≥15 | Low |
| 123 | (Johansen et al., 2018) [122] | 212 | 281 | 162 | 57 | 18-86 | 2018 | Norway | Europe | CS | Mixed | Mixed | Under/Treat | GSDS | ≥ 43 | Moderate |
| 124 | (Lowery-Allison et al., 2018) [123] | 76 | 200 | 200 | 57 |  | 2018 | US | North America | CS | Breast | No Evidence of Disease | > 3 month After/Treat | PSQI | ˃8 | Moderate |
| 125 | (Overcash et al., 2018) [124] | 20 | 60 | 60 | 77.6 | 69-93 | 2018 | US | North America | CS | Breast | Mixed | Under/Treat | PSQI | ˃5 | Moderate |
| 126 | (Palesh et al., 2018) [125] | 36 | 71 | 71 | 52.5 |  | 2018 | US | North America | LG | Mixed | Localized | Under/Treat | ISI | ˃8 | Moderate |
| 127 | (Saberzadeh-Ardestani et al., 2019) [126] | 16 | 74 | 42 | 68.9 |  | 2019 | Iran | Asia | LG | Breast | Mixed | > 3 month After/Treat | ESS | ≥10 | Moderate |
| 128 | (Berger et al., 2019) [127] | 690 | 1302 | 1302 | 60.1 | 21-90 | 2019 | US | North America | CS | Breast | Localized | > 3 month After/Treat | PSQI | ˃5 | Low |
| 129 | (Li et al., 2019) [128] | 137 | 180 | 180 | 53 | 22-74 | 2019 | China | Asia | LG | Breast | Localized | Under/Treat | PSQI | ˃5 | Moderate |
| 130 | (Tejada et al., 2019) [129] | 998 | 1331 | 1037 | 57.1 |  | 2019 | US | North America | LG | Mixed | Mixed | Under/Treat | GSDS | ≥ 43 | Low |
| 131 | (Sun et al., 2020) [130] | 166 | 434 | 180 | 62.6 | 25-89 | 2019 | China | Asia | CS | Gastrointestinal | Mixed | Under/Treat | AIS | ≥6 | Moderate |
| 132 | (Papadopoulos et al., 2019) [131] | 70 | 119 | 34 | 64 |  | 2019 | Greek | Europe | CS | Lung | Mixed | > 3 month After/Treat | PSQI | ˃5 | Moderate |
| 133 | (Zubair et al., 2019) [132] | 112 | 170 | 60 | 52.1 |  | 2019 | Pakistan | Asia | CS | Head and Neck | Mixed | > 3 month After/Treat | PSQI | ˃5 | Moderate |
| 134 | (Imanian et al., 2019) [133] | 114 | 115 | 115 | 47 | 25-69 | 2019 | Iran | Asia | CS | Breast | Localized | Under/Treat | PSQI | ˃5 | Moderate |
| 135 | (Schieber et al., 2019) [134] | 260 | 405 | 227 | 58.6 | 20-90 | 2019 | Germany | Europe | LG | Mixed | Mixed | Under/Treat | ISI | ≥7 | Moderate |
| 136 | (Liou et al., 2019) [135] | 556 | 1072 | 1072 | 62.1 |  | 2019 | US | North America | CS | Breast | Localized | > 3 month After/Treat | ISI | ≥8 | Low |
| 137 | (Fleming et al., 2019) [136] | 97 | 173 | 173 | 58 |  | 2019 | UK | Europe | LG | Breast | Localized | Under/Treat | ISI | ≥7 | Low |
| 138 | (Fong and Ho, 2020) [137] | 71 | 127 | 74 | 68 |  | 2019 | Hong Kong | Asia | LG | Gastrointestinal | Localized | Under/Treat | PSQI | ≥8 | Moderate |
| 139 | (Garland et al., 2019) [138] | 157 | 160 | 91 | 61.5 |  | 2019 | US | North America | LG | Mixed | No Evidence of Disease | > 3 month After/Treat | ISI | ˃8 | Moderate |
| 140 | (Strollo et al., 2020) [139] | 968 | 1903 | 1193 | 64.5 |  | 2020 | US | North America | CS | Mixed | Localized | > 3 month After/Treat | PSQI | ˃5 | Moderate |
| 141 | (Yoshikawa et al., 2020) [140] | 70 | 139 | 42 | 64 | 35-89 | 2020 | Japan | Asia | CS | Gastrointestinal | Mixed | Under/Treat | PSQI | ˃5 | Moderate |
| 142 | (Saesen et al., 2021) [141] | 20 | 50 | 17 | 64.2 | 32-88 | 2020 | Belgium | Europe | LG | Head and Neck | Mixed | Under/Treat | ESS | ≥10 | Moderate |
| 143 | (İzci et al., 2020) [142] | 23 | 56 | 56 | 53 |  | 2020 | Turkey | Asia | CS | Breast | Localized | > 3 month After/Treat | PSQI | ˃5 | Moderate |
| 144 | (ŞANLIER et al., 2020) [143] | 144 | 264 | 132 | 57.3 | 19-88 | 2020 | Turkey | Asia | CS | Breast | Localized | Under/Treat | PSQI | ˃5 | Moderate |
| 145 | (Al Maqbali et al., 2020) [144] | 288 | 369 | 245 |  | 20-75 | 2020 | Oman | Asia | CS | Mixed | Mixed | > 3 month After/Treat | PSQI | ˃5 | Moderate |
| 146 | (Fekih-Romdhane et al., 2020) [145] | 33 | 50 | 50 | 52 | 34-65 | 2020 | Tunis | Africa | CS | Breast | Mixed | Under/Treat | PSQI | ˃5 | Moderate |
| 147 | (Pai et al., 2020) [146] | 53 | 92 | 67 | 53 | 18-79 | 2020 | India | Asia | CS | Mixed | Mixed | Under/Treat | PSQI | ˃5 | Moderate |
| 148 | (Hoang et al., 2020) [147] | 91 | 213 | 129 | 53.1 |  | 2020 | Vietnam | Asia | CS | Mixed | Mixed | Under/Treat | ISI | ≥7 | Moderate |
| 149 | (Chan et al., 2020) [148] | 60 | 98 | 98 | 51.4 |  | 2020 | Taiwan | Asia | CS | Breast | No Evidence of Disease | > 3 month After/Treat | PSQI | ˃5 | Moderate |
| 150 | (Lin et al., 2020b) [149] | 438 | 573 | 247 | 61.3 |  | 2020 | Iran | Asia | LG | Mixed | advanced | Mixed | ISI | ˃8 | Moderate |
| 151 | (Lin et al., 2020a) [150] | 773 | 859 | 348 | 67.4 |  | 2020 | Iran | Asia | LG | Mixed | advanced | > 3 month After/Treat | PSQI | ˃5 | Low |
| 152 | (Martin et al., 2020) [151] | 37 | 64 | 64 | 57.8 | 22-75 | 2020 | US | North America | CS | Breast | No Evidence of Disease | > 3 month After/Treat | ISI | ˃8 | Moderate |
| 153 | (Gonzalez et al., 2021) [152] | 451 | 632 | 632 | 55 |  | 2021 | US | North America | LG | Breast | Mixed | > 3 month After/Treat | PSQI | ≥5 | Moderate |
| 154 | (Santoso et al., 2021) [153] | 246 | 560 | 142 | 63 |  | 2021 | Netherland | Europe | CS | Head and Neck | Mixed | Under/Treat | PSQI | ˃5 | Moderate |
| 155 | (Al Maqbali, 2021) [154] | 98 | 133 | 133 |  | 20-75 | 2021 | Oman | Asia | CS | Mixed | Mixed | > 3 month After/Treat | PSQI | ˃5 | Moderate |
| 156 | (Kreutz et al., 2021) [155] | 28 | 54 | 54 | 49 |  | 2021 | Germany | Europe | CS | Mixed | Mixed | > 3 month After/Treat | PSQI | ˃5 | Moderate |
| 157 | (Ratcliff et al., 2021) [156] | 200 | 375 | 375 | 49.82 |  | 2021 | US | North America | LG | Breast | Localized | Under/Treat | PSQI | ˃5 | Moderate |
| 158 | (Daldoul et al., 2021) [157] | 28 | 70 | 70 | 43 |  | 2021 | Tunis | Africa | CS | Breast | Mixed | Under/Treat | ISI | ˃8 | Moderate |
| 159 | (Nguyen et al., 2021) [158] | 61 | 80 | 80 | 62 |  | 2021 | Australia | Australia | LG | Breast | No Evidence of Disease | > 3 month After/Treat | PSQI | ˃5 | Moderate |
| 160 | (Jeon et al., 2021) [159] | 43 | 81 | 31 | 51.1 |  | 2021 | Australia | Australia | CS | Brain | Mixed | Mixed | PSQI | ˃5 | Moderate |

CS= Cross Sectional; LG= Longitudinal; Athens Insomnia Scale (AIS); Epworth Sleepiness Scale (ESS); General Sleep Disturbance Scale (GSDS); Hamilton Depression Inventory (HDI); Insomnia Severity Index (ISI); Medical Outcomes Study-Sleep Scale (MOS-SS); Pittsburgh Sleep Quality Index (PSQI); Sleep Disturbance Adult Scale (SDAS); Sleep Impairment Index (SII); Sleep Questionnaire (SQ); Women's Health Initiative- -Sleep Disturbance Scale (WHI-SDS).

**References**

1. Carpenter JS, Andrykowski MA (1998) Psychometric evaluation of the Pittsburgh sleep quality index. Journal of psychosomatic research 45:5–13

2. Savard J, Simard S, Blanchet J, et al (2001) Prevalence, clinical characteristics, and risk factors for insomnia in the context of breast cancer. Sleep 24:583–590. https://doi.org/10.1093/sleep/24.5.583

3. Fortner BV, Stepanski EJ, Wang SC, et al (2002) Sleep and quality of life in breast cancer patients. J Pain Symptom Manage 24:471–480. https://doi.org/10.1016/s0885-3924(02)00500-6

4. Beck SL, Schwartz AL, Towsley G, et al (2004) Psychometric evaluation of the Pittsburgh sleep quality index in cancer patients. J Pain Symptom Manage 27:140–148. https://doi.org/10.1016/j.jpainsymman.2003.12.002

5. Savard M-H, Savard J, Simard S, Ivers H (2005) Empirical validation of the Insomnia Severity Index in cancer patients. Psycho-Oncology 14:429–441. https://doi.org/10.1002/pon.860

6. Savard J, Simard S, Hervouet S, et al (2005) Insomnia in men treated with radical prostatectomy for prostate cancer. Psychooncology 14:147–156. https://doi.org/10.1002/pon.830

7. Sela RA, Watanabe S, Nekolaichuk CL (2005) Sleep disturbances in palliative cancer patients attending a pain and symptom control clinic. Palliat Support Care 3:23–31. https://doi.org/10.1017/s1478951505050042

8. Carlson LE, Garland SN (2005) Impact of mindfulness-based stress reduction (MBSR) on sleep, mood, stress and fatigue symptoms in cancer outpatients. Int J Behav Med 12:278–285. https://doi.org/10.1207/s15327558ijbm1204_9

9. Ancoli-Israel S, Liu L, Marler MR, et al (2006) Fatigue, sleep, and circadian rhythms prior to chemotherapy for breast cancer. Support Care Cancer 14:201–209. https://doi.org/10.1007/s00520-005-0861-0

10. Gooneratne NS, Dean GE, Rogers AE, et al (2007) Sleep and quality of life in long-term lung cancer survivors. Lung Cancer 58:403–410. https://doi.org/10.1016/j.lungcan.2007.07.011

11. Berger AM, Farr LA, Kuhn BR, et al (2007) Values of sleep/wake, activity/rest, circadian rhythms, and fatigue prior to adjuvant breast cancer chemotherapy. J Pain Symptom Manage 33:398–409. https://doi.org/10.1016/j.jpainsymman.2006.09.022

12. Fox SW, Lyon D, Farace E (2007) Symptom Clusters in Patients With High-Grade Glioma. Journal of Nursing Scholarship 39:61–67. https://doi.org/10.1111/j.1547-5069.2007.00144.x

13. Palesh OG, Collie K, Batiuchok D, et al (2007) A Longitudinal Study of Depression, Pain, and Stress as Predictors of Sleep Disturbance among Women with Metastatic Breast Cancer. Biol Psychol 75:37–44. https://doi.org/10.1016/j.biopsycho.2006.11.002

14. Akechi T, Okuyama T, Akizuki N, et al (2007) Associated and predictive factors of sleep disturbance in advanced cancer patients. Psycho-Oncology 16:888–894

15. Mystakidou K, Parpa E, Tsilika E, et al (2007) Sleep quality in advanced cancer patients. J Psychosom Res 62:527–533. https://doi.org/10.1016/j.jpsychores.2006.11.008

16. Shahidi J, Khodabakhshi R, Yahyazadeh SH, et al (2007) Quality of sleep in cancer patients: evidence from Persian translation of Pittsburg Sleep Quality Index. Austral Asian J Cancer 6:165–8

17. Bardwell WA, Profant J, Casden DR, et al (2008) The relative importance of specific risk factors for insomnia in women treated for early-stage breast cancer. Psychooncology 17:9–18. https://doi.org/10.1002/pon.1192

18. Palesh O, Zeitzer JM, Conrad A, et al (2008) Vagal Regulation, Cortisol, and Sleep Disruption in Women with Metastatic Breast Cancer. J Clin Sleep Med 4:441–449

19. Rogers LQ, Courneya KS, Robbins KT, et al (2008) Factors associated with fatigue, sleep, and cognitive function among patients with head and neck cancer. Head Neck 30:1310–1317. https://doi.org/10.1002/hed.20873

20. Chen M-L, Yu C-T, Yang C-H (2008) Sleep disturbances and quality of life in lung cancer patients undergoing chemotherapy. Lung Cancer 62:391–400. https://doi.org/10.1016/j.lungcan.2008.03.016

21. Dirksen SR, Belyea MJ, Epstein DR (2009) Fatigue-based subgroups of breast cancer survivors with insomnia. Cancer Nurs 32:404–411. https://doi.org/10.1097/NCC.0b013e3181a5d05e

22. Tremblay V, Savard J, Ivers H (2009) Predictors of the effect of cognitive behavioral therapy for chronic insomnia comorbid with breast cancer. J Consult Clin Psychol 77:742–750. https://doi.org/10.1037/a0015492

23. Liu L, Fiorentino L, Natarajan L, et al (2009) Pre-treatment symptom cluster in breast cancer patients is associated with worse sleep, fatigue and depression during chemotherapy. Psychooncology 18:187–194. https://doi.org/10.1002/pon.1412

24. Dirksen SR, Epstein DR, Hoyt MA (2009) Insomnia, depression, and distress among outpatients with prostate cancer. Appl Nurs Res 22:154–158. https://doi.org/10.1016/j.apnr.2007.09.001

25. Gibbins J, McCoubrie R, Kendrick AH, et al (2009) Sleep-wake disturbances in patients with advanced cancer and their family carers. J Pain Symptom Manage 38:860–870. https://doi.org/10.1016/j.jpainsymman.2009.04.025

26. Mystakidou K, Parpa E, Tsilika E, et al (2009) How is sleep quality affected by the psychological and symptom distress of advanced cancer patients? Palliat Med 23:46–53. https://doi.org/10.1177/0269216308098088

27. Aouizerat BE, Dodd M, Lee K, et al (2009) Preliminary evidence of a genetic association between tumor necrosis factor alpha and the severity of sleep disturbance and morning fatigue. Biol Res Nurs 11:27–41. https://doi.org/10.1177/1099800409333871

28. Price MA, Zachariae R, Butow PN, et al (2009) Prevalence and predictors of insomnia in women with invasive ovarian cancer: Anxiety a major factor. European Journal of Cancer 45:3262–3270. https://doi.org/10.1016/j.ejca.2009.05.030

29. Savard J, Villa J, Ivers H, et al (2009) Prevalence, natural course, and risk factors of insomnia comorbid with cancer over a 2-month period. J Clin Oncol 27:5233–5239. https://doi.org/10.1200/JCO.2008.21.6333

30. Berger AM, Kuhn BR, Farr LA, et al (2009) One-year outcomes of a behavioral therapy intervention trial on sleep quality and cancer-related fatigue. J Clin Oncol 27:6033–6040. https://doi.org/10.1200/JCO.2008.20.8306

31. Palesh OG, Roscoe JA, Mustian KM, et al (2010) Prevalence, demographics, and psychological associations of sleep disruption in patients with cancer: University of Rochester Cancer Center-Community Clinical Oncology Program. J Clin Oncol 28:292–298. https://doi.org/10.1200/JCO.2009.22.5011

32. Van Onselen C, Dunn LB, Lee K, et al (2010) Relationship between mood disturbance and sleep quality in oncology outpatients at the initiation of radiation therapy. Eur J Oncol Nurs 14:373–379. https://doi.org/10.1016/j.ejon.2009.12.002

33. Vargas S, Wohlgemuth WK, Antoni MH, et al (2010) Brief Report: Sleep Dysfunction and Psychosocial Adaptation Among Women Undergoing Treatment for Non-Metastatic Breast Cancer. Psychooncology 19:669–673. https://doi.org/10.1002/pon.1603

34. Otte JL, Carpenter JS, Russell KM, et al (2010) Prevalence, Severity, and Correlates of Sleep-Wake Disturbances in Long-Term Breast Cancer Survivors. J Pain Symptom Manage 39:535–547. https://doi.org/10.1016/j.jpainsymman.2009.07.004

35. Dodd MJ, Cho MH, Cooper BA, Miaskowski C (2010) The effect of symptom clusters on functional status and quality of life in women with breast cancer. Eur J Oncol Nurs 14:101–110. https://doi.org/10.1016/j.ejon.2009.09.005

36. Barsevick A, Beck SL, Dudley WN, et al (2010) Efficacy of an intervention for fatigue and sleep disturbance during cancer chemotherapy. J Pain Symptom Manage 40:200–216. https://doi.org/10.1016/j.jpainsymman.2009.12.020

37. Beck SL, Berger AM, Barsevick AM, et al (2010) Sleep quality after initial chemotherapy for breast cancer. Support Care Cancer 18:679–689. https://doi.org/10.1007/s00520-009-0662-y

38. Park JH, Lee SJ, Gwak JI, et al (2010) Sleep Quality of Breast Cancer Patients Receiving Chemotherapy in the Outpatients Setting. Korean Journal of Family Medicine 31:778–785

39. Garrett K, Dhruva A, Koetters T, et al (2011) Differences in Sleep Disturbance and Fatigue Between Patients with Breast and Prostate Cancer at the Initiation of Radiation Therapy. Journal of Pain and Symptom Management 42:239–250. https://doi.org/10.1016/j.jpainsymman.2010.11.010

40. Miaskowski C, Paul SM, Cooper BA, et al (2011) Predictors of the trajectories of self-reported sleep disturbance in men with prostate cancer during and following radiation therapy. Sleep 34:171–179. https://doi.org/10.1093/sleep/34.2.171

41. Miaskowski C, Lee K, Dunn L, et al (2011) Sleep-Wake Circadian Activity Rhythm Parameters and Fatigue in Oncology Patients Prior to the Initiation of Radiation Therapy. Cancer Nurs 34:255–268. https://doi.org/10.1097/NCC.0b013e3181f65d9b

42. Sandadi S, Frasure HE, Broderick MJ, et al (2011) The effect of sleep disturbance on quality of life in women with ovarian cancer. Gynecol Oncol 123:351–355. https://doi.org/10.1016/j.ygyno.2011.07.028

43. Delgado-Guay M, Yennurajalingam S, Parsons H, et al (2011) Association between self-reported sleep disturbance and other symptoms in patients with advanced cancer. J Pain Symptom Manage 41:819–827. https://doi.org/10.1016/j.jpainsymman.2010.07.015

44. Bower JE, Ganz PA, Irwin MR, et al (2011) Inflammation and Behavioral Symptoms After Breast Cancer Treatment: Do Fatigue, Depression, and Sleep Disturbance Share a Common Underlying Mechanism? Journal of Clinical Oncology 29:3517–3522. https://doi.org/10.1200/JCO.2011.36.1154

45. Hanisch LJ, Gooneratne NS, Soin K, et al (2011) Sleep and daily functioning during androgen deprivation therapy for prostate cancer. Eur J Cancer Care (Engl) 20:549–554. https://doi.org/10.1111/j.1365-2354.2010.01226.x

46. Colagiuri B, Christensen S, Jensen AB, et al (2011) Prevalence and Predictors of Sleep Difficulty in a National Cohort of Women With Primary Breast Cancer Three to Four Months Postsurgery. Journal of Pain and Symptom Management 42:710–720. https://doi.org/10.1016/j.jpainsymman.2011.02.012

47. Moore TA, Berger AM, Dizona P (2011) Sleep aid use during and following breast cancer adjuvant chemotherapy. Psychooncology 20:321–325. https://doi.org/10.1002/pon.1756

48. Grutsch JF, Wood PA, Du-Quiton J, et al (2011) Validation of actigraphy to assess circadian organization and sleep quality in patients with advanced lung cancer. J Circadian Rhythms 9:4. https://doi.org/10.1186/1740-3391-9-4

49. Sun J-L, Chiou J-F, Lin C-C (2011) Validation of the Taiwanese version of the Athens Insomnia Scale and assessment of insomnia in Taiwanese cancer patients. J Pain Symptom Manage 41:904–914. https://doi.org/10.1016/j.jpainsymman.2010.07.021

50. Rand KL, Otte JL, Flockhart D, et al (2011) Modeling hot flushes and quality of life in breast cancer survivors. Climacteric 13:171–180. https://doi.org/10.3109/13697131003717070

51. Kotronoulas GC, Papadopoulou CN, Papapetrou A, Patiraki E (2011) Psychometric evaluation and feasibility of the Greek Pittsburgh Sleep Quality Index (GR-PSQI) in patients with cancer receiving chemotherapy. Supportive Care in Cancer 19:1831–1840. https://doi.org/10.1007/s00520-010-1025-4

52. Enderlin CA, Coleman EA, Cole C, et al (2011) Subjective sleep quality, objective sleep characteristics, insomnia symptom severity, and daytime sleepiness in women aged 50 and older with nonmetastatic breast cancer. Oncol Nurs Forum 38:E314-325. https://doi.org/10.1188/11.ONF.E314-E325

53. Dhruva A, Paul SM, Cooper BA, et al (2012) A longitudinal study of measures of objective and subjective sleep disturbance in patients with breast cancer before, during, and after radiation therapy. Journal of Pain and Symptom Management 44:215–228

54. Phillips KM, Jim HS, Donovan KA, et al (2012) Characteristics and correlates of sleep disturbances in cancer patients. Support Care Cancer 20:357–365. https://doi.org/10.1007/s00520-011-1106-z

55. Forsythe LP, Helzlsouer KJ, MacDonald R, Gallicchio L (2012) Daytime sleepiness and sleep duration in long-term cancer survivors and non-cancer controls: results from a registry-based survey study. Support Care Cancer 20:2425–2432. https://doi.org/10.1007/s00520-011-1358-7

56. Liu L, Rissling M, Natarajan L, et al (2012) The longitudinal relationship between fatigue and sleep in breast cancer patients undergoing chemotherapy. Sleep 35:237–245. https://doi.org/10.5665/sleep.1630

57. Mosher CE, Duhamel KN (2012) An examination of distress, sleep, and fatigue in metastatic breast cancer patients. Psychooncology 21:100–107. https://doi.org/10.1002/pon.1873

58. Liu L, Mills PJ, Rissling M, et al (2012) Fatigue and Sleep Quality are Associated with Changes in Inflammatory Markers in Breast Cancer Patients Undergoing Chemotherapy. Brain Behav Immun 26:706–713. https://doi.org/10.1016/j.bbi.2012.02.001

59. Clevenger L, Schrepf A, Christensen D, et al (2012) Sleep disturbance, cytokines, and fatigue in women with ovarian cancer. Brain Behav Immun 26:1037–1044. https://doi.org/10.1016/j.bbi.2012.04.003

60. Van Onselen C, Cooper BA, Lee K, et al (2012) Identification of distinct subgroups of breast cancer patients based on self-reported changes in sleep disturbance. Support Care Cancer 20:2611–2619. https://doi.org/10.1007/s00520-012-1381-3

61. Mansano-Schlosser TC, Ceolim MF (2012) Factors associated with sleep quality in the elderly receiving chemotherapy. Rev Lat Am Enfermagem 20:1100–1108. https://doi.org/10.1590/s0104-11692012000600012

62. Taylor TR, Huntley ED, Makambi K, et al (2012) Understanding sleep disturbances in African-American breast cancer survivors: a pilot study. Psychooncology 21:896–902. https://doi.org/10.1002/pon.2000

63. Tzeng JI, Fu Y-W, Lin C-C (2012) Validity and reliability of the Taiwanese version of the Pittsburgh Sleep Quality Index in cancer patients. International Journal of Nursing Studies 49:102–108. https://doi.org/10.1016/j.ijnurstu.2011.08.004

64. Desai K, Mao JJ, Su I, et al (2013) Prevalence and risk factors for insomnia among breast cancer patients on aromatase inhibitors. Support Care Cancer 21:43–51. https://doi.org/10.1007/s00520-012-1490-z

65. Clevenger L, Schrepf A, Degeest K, et al (2013) Sleep disturbance, distress, and quality of life in ovarian cancer patients during the first year after diagnosis. Cancer 119:3234–3241. https://doi.org/10.1002/cncr.28188

66. Sanford SD, Wagner LI, Beaumont JL, et al (2013) Longitudinal prospective assessment of sleep quality: before, during, and after adjuvant chemotherapy for breast cancer. Support Care Cancer 21:959–967. https://doi.org/10.1007/s00520-012-1612-7

67. Saini A, Berruti A, Ferini-Strambi L, et al (2013) Restless Legs Syndrome as a Cause of Sleep Disturbances in Cancer Patients Receiving Chemotherapy. Journal of Pain and Symptom Management 46:56–64. https://doi.org/10.1016/j.jpainsymman.2012.06.018

68. Caplette-Gingras A, Savard J, Savard M-H, Ivers H (2013) Is insomnia associated with cognitive impairments in breast cancer patients? Behav Sleep Med 11:239–257. https://doi.org/10.1080/15402002.2012.672940

69. Nakamura Y, Lipschitz DL, Kuhn R, et al (2013) Investigating efficacy of two brief mind-body intervention programs for managing sleep disturbance in cancer survivors: a pilot randomized controlled trial. J Cancer Surviv 7:165–182. https://doi.org/10.1007/s11764-012-0252-8

70. Hall DL, Mishel MH, Germino BB (2014) Living with cancer-related uncertainty: associations with fatigue, insomnia, and affect in younger breast cancer survivors. Support Care Cancer 22:2489–2495. https://doi.org/10.1007/s00520-014-2243-y

71. Doong S-H, Dhruva A, Dunn LB, et al (2015) Associations Between Cytokine Genes and a Symptom Cluster of Pain, Fatigue, Sleep Disturbance, and Depression in Patients Prior to Breast Cancer Surgery. Biol Res Nurs 17:237–247. https://doi.org/10.1177/1099800414550394

72. Ho RTH, Fong TCT (2014) Factor structure of the Chinese version of the Pittsburgh Sleep Quality Index in breast cancer patients. Sleep Medicine 15:565–569. https://doi.org/10.1016/j.sleep.2013.10.019

73. Ma C-L, Chang W-P, Lin C-C (2014) Rest/activity rhythm is related to the coexistence of pain and sleep disturbance among advanced cancer patients with pain. Support Care Cancer 22:87–94. https://doi.org/10.1007/s00520-013-1918-0

74. Hong JS, Tian J, Wu LH (2014) The influence of chemotherapy-induced neurotoxicity on psychological distress and sleep disturbance in cancer patients. Curr Oncol 21:174–180. https://doi.org/10.3747/co.21.1984

75. Mao JJ, Farrar JT, Bruner D, et al (2014) Electroacupuncture for fatigue, sleep, and psychological distress in breast cancer patients with aromatase inhibitor-related arthralgia: a randomized trial. Cancer 120:3744–3751. https://doi.org/10.1002/cncr.28917

76. Romito F, Cormio C, De Padova S, et al (2014) Patients attitudes towards sleep disturbances during chemotherapy. Eur J Cancer Care (Engl) 23:385–393. https://doi.org/10.1111/ecc.12106

77. Vargas S, Antoni MH, Carver CS, et al (2014) Sleep quality and fatigue after a stress management intervention for women with early-stage breast cancer in southern Florida. Int J Behav Med 21:971–981. https://doi.org/10.1007/s12529-013-9374-2

78. Courneya KS, Segal RJ, Mackey JR, et al (2014) Effects of exercise dose and type on sleep quality in breast cancer patients receiving chemotherapy: a multicenter randomized trial. Breast Cancer Res Treat 144:361–369. https://doi.org/10.1007/s10549-014-2883-0

79. Kashani F, Kashani P (2014) The effect of massage therapy on the quality of sleep in breast cancer patients. Iran J Nurs Midwifery Res 19:113–118

80. Valko PO, Siddique A, Linsenmeier C, et al (2015) Prevalence and predictors of fatigue in glioblastoma: a prospective study. Neuro Oncol 17:274–281. https://doi.org/10.1093/neuonc/nou127

81. Ho S-Y, Rohan KJ, Parent J, et al (2015) A Longitudinal Study of Depression, Fatigue, and Sleep Disturbances as a Symptom Cluster in Women with Breast Cancer. J Pain Symptom Manage 49:707–715. https://doi.org/10.1016/j.jpainsymman.2014.09.009

82. Klyushnenkova EN, Sorkin JD, Gallicchio L (2015) Association of obesity and sleep problems among breast cancer survivors: results from a registry-based survey study. Support Care Cancer 23:3437–3445. https://doi.org/10.1007/s00520-015-2692-y

83. Mercadante S, Aielli F, Adile C, et al (2015) Sleep Disturbances in Patients With Advanced Cancer in Different Palliative Care Settings. J Pain Symptom Manage 50:786–792. https://doi.org/10.1016/j.jpainsymman.2015.06.018

84. He Y, Meng Z, Jia Q, et al (2015) Sleep Quality of Patients with Differentiated Thyroid Cancer. PLOS ONE 10:e0130634. https://doi.org/10.1371/journal.pone.0130634

85. Nishiura M, Tamura A, Nagai H, Matsushima E (2015) Assessment of sleep disturbance in lung cancer patients: Relationship between sleep disturbance and pain, fatigue, quality of life, and psychological distress. Palliative & Supportive Care 13:575–581. https://doi.org/10.1017/S1478951513001119

86. Yu S-Y, Nho J-H (2015) Influence of Sleep Disturbance and Depression on Quality of Life in Ovarian Cancer Patients during Chemotherapy. Asian Oncology Nursing 15:203–210. https://doi.org/10.5388/aon.2015.15.4.203

87. Qin L, Mo Y-L, Li L, et al (2015) Sleep characteristics and psychological symptoms in patients with locally advanced nasopharyngeal carcinoma before and after intensity-modulated radiotherapy and concurrent chemotherapy. Psychology, Health & Medicine 20:662–669. https://doi.org/10.1080/13548506.2014.967703

88. Morris BA, Thorndike FP, Ritterband LM, et al (2015) Sleep disturbance in cancer patients and caregivers who contact telephone-based help services. Support Care Cancer 23:1113–1120. https://doi.org/10.1007/s00520-014-2458-y

89. Tian J, Chen GL, Zhang HR (2015) Sleep status of cervical cancer patients and predictors of poor sleep quality during adjuvant therapy. Support Care Cancer 23:1401–1408. https://doi.org/10.1007/s00520-014-2493-8

90. Savard J, Ivers H, Savard M-H, Morin CM (2015) Cancer treatments and their side effects are associated with aggravation of insomnia: Results of a longitudinal study. Cancer 121:1703–1711. https://doi.org/10.1002/cncr.29244

91. Akman T, Yavuzsen T, Sevgen Z, et al (2015) Evaluation of sleep disorders in cancer patients based on Pittsburgh Sleep Quality Index. European Journal of Cancer Care 24:553–559

92. Berrett-Abebe J, Cadet T, Pirl W, Lennes I (2015) Exploring the relationship between fear of cancer recurrence and sleep quality in cancer survivors. J Psychosoc Oncol 33:297–309. https://doi.org/10.1080/07347332.2015.1020586

93. Roscoe JA, Garland SN, Heckler CE, et al (2015) Randomized placebo-controlled trial of cognitive behavioral therapy and armodafinil for insomnia after cancer treatment. J Clin Oncol 33:165–171. https://doi.org/10.1200/JCO.2014.57.6769

94. Lafçi D, Öztunç G (2015) The effect of music on the sleep quality of breast cancer patients. International journal of caring sciences 8:633

95. Li C-C, Tsai Y-F, Chang T-C, Chen L (2017) Associations among menopausal symptoms, sleep and fatigue in Taiwanese women with endometrial cancer. Eur J Cancer Care (Engl) 26:. https://doi.org/10.1111/ecc.12559

96. Jung D, Lee K-M, Kim W-H, et al (2016) Longitudinal Association of Poor Sleep Quality With Chemotherapy-Induced Nausea and Vomiting in Patients With Breast Cancer. Psychosomatic Medicine 78:959. https://doi.org/10.1097/PSY.0000000000000372

97. Collins KP, Geller DA, Antoni M, et al (2017) Sleep duration is associated with survival in advanced cancer patients. Sleep Med 32:208–212. https://doi.org/10.1016/j.sleep.2016.06.041

98. Bagheri-Nesami M, Goudarzian AH, Jan Babaei G, et al (2016) Sleep Quality and Associated Risk Factors in Leukemia Patients Undergoing Chemotherapy in Iran. Asian Pac J Cancer Prev 17:107–111. https://doi.org/10.7314/apjcp.2016.17.s3.107

99. Fontes F, Gonçalves M, Pereira S, Lunet N (2017) Neuropathic pain after breast cancer treatment and its impact on sleep quality one year after cancer diagnosis. The Breast 33:125–131. https://doi.org/10.1016/j.breast.2017.03.013

100. Loth A, Michel J, Giorgi R, et al (2017) Prevalence of obstructive sleep apnoea syndrome following oropharyngeal cancer treatment: A prospective cohort study. Clin Otolaryngol 42:1281–1288. https://doi.org/10.1111/coa.12869

101. Mercadante S, Adile C, Ferrera P, et al (2017) Sleep disturbances in advanced cancer patients admitted to a supportive/palliative care unit. Support Care Cancer 25:1301–1306. https://doi.org/10.1007/s00520-016-3524-4

102. Halle IH, Westgaard TK, Wahba A, et al (2017) Trajectory of sleep disturbances in patients undergoing lung cancer surgery: a prospective study. Interact CardioVasc Thorac Surg 25:285–291. https://doi.org/10.1093/icvts/ivx076

103. Chung S, Youn S, Choi B (2017) Assessment of Cancer-Related Dysfunctional Beliefs about Sleep for Evaluating Sleep Disturbance in Cancer Patients. Sleep Med Res 8:98–101. https://doi.org/10.17241/smr.2017.00073

104. Ji Y-B, Bo C-L, Xue X-J, et al (2017) Association of Inflammatory Cytokines With the Symptom Cluster of Pain, Fatigue, Depression, and Sleep Disturbance in Chinese Patients With Cancer. J Pain Symptom Manage 54:843–852. https://doi.org/10.1016/j.jpainsymman.2017.05.003

105. Davies AN, Patel SD, Gregory A, Lee B (2017) Observational study of sleep disturbances in advanced cancer. BMJ Support Palliat Care 7:435–440. https://doi.org/10.1136/bmjspcare-2017-001363

106. Echchikhi Y, El-Abbassi S, Touil A, et al (2017) Sleep disorders and sleep quality in Moroccan adult patients with cancer during treatment. J Cancer Sci Ther 9:637–643

107. Rogers LQ, Courneya KS, Oster RA, et al (2017) Physical Activity and Sleep Quality in Breast Cancer Survivors: A Randomized Trial. Med Sci Sports Exerc 49:2009–2015. https://doi.org/10.1249/MSS.0000000000001327

108. Cha KM, Chung YK, Lim KY, et al (2017) Depression and insomnia as mediators of the relationship between distress and quality of life in cancer patients. Journal of Affective Disorders 217:260–265. https://doi.org/10.1016/j.jad.2017.04.020

109. Peoples AR, Garland SN, Perlis ML, et al (2017) Effects of cognitive behavioral therapy for insomnia and armodafinil on quality of life in cancer survivors: a randomized placebo-controlled trial. J Cancer Surviv 11:401–409. https://doi.org/10.1007/s11764-017-0597-0

110. Mansano-Schlosser TC, Ceolim MF, Valerio TD (2017) Poor sleep quality, depression and hope before breast cancer surgery. Appl Nurs Res 34:7–11. https://doi.org/10.1016/j.apnr.2016.11.010

111. Li N, Otomaru T, Taniguchi H (2017) Sleep quality in long-term survivors of head and neck cancer: preliminary findings. Support Care Cancer 25:3741–3748. https://doi.org/10.1007/s00520-017-3804-7

112. Miladinia M, Baraz S, Ramezani M, Malehi AS (2018) The relationship between pain, fatigue, sleep disorders and quality of life in adult patients with acute leukaemia: During the first year after diagnosis. European Journal of Cancer Care 27:e12762. https://doi.org/10.1111/ecc.12762

113. Gu F, Li X-F, Xu J-F, et al (2018) Effect of nicotine dependence on quality of life and sleep quality in patients with lung cancer who continue to smoke after diagnosis. J Thorac Dis 10:2583–2589. https://doi.org/10.21037/jtd.2018.05.12

114. Adams SC, DeLorey DS, Davenport MH, et al (2018) Effects of high-intensity interval training on fatigue and quality of life in testicular cancer survivors. Br J Cancer 118:1313–1321. https://doi.org/10.1038/s41416-018-0044-7

115. Steel JL, Terhorst L, Collins KP, et al (2018) Prospective Analyses of Cytokine Mediation of Sleep and Survival in the Context of Advanced Cancer. Psychosom Med 80:483–491. https://doi.org/10.1097/PSY.0000000000000579

116. Henneghan AM, Carter P, Stuifbergan A, et al (2018) Relationships between self-reported sleep quality components and cognitive functioning in breast cancer survivors up to 10 years following chemotherapy. Psycho-Oncology 27:1937–1943. https://doi.org/10.1002/pon.4745

117. Jim HSL, Sutton S, Majhail NS, et al (2018) Severity, course, and predictors of sleep disruption following hematopoietic cell transplantation: a secondary data analysis from the BMT CTN 0902 trial. Bone Marrow Transplant 53:1038–1043. https://doi.org/10.1038/s41409-018-0138-0

118. Nelson AM, Jim HSL, Small BJ, et al (2018) Sleep Disruption Among Cancer Patients Following Autologous Hematopoietic Cell Transplantation. Bone Marrow Transplant 53:307–314. https://doi.org/10.1038/s41409-017-0022-3

119. Gonzalez BD, Small BJ, Cases MG, et al (2018) Sleep disturbance in men receiving androgen deprivation therapy for prostate cancer: The role of hot flashes and nocturia. Cancer 124:499–506. https://doi.org/10.1002/cncr.31024

120. Dreher M, Krüger S, Schulze-Olden S, et al (2018) Sleep-disordered breathing in patients with newly diagnosed lung cancer. BMC Pulm Med 18:72. https://doi.org/10.1186/s12890-018-0645-1

121. Mao H, Bao T, Shen X, et al (2018) Prevalence and risk factors for fatigue among breast cancer survivors on aromatase inhibitors. Eur J Cancer 101:47–54. https://doi.org/10.1016/j.ejca.2018.06.009

122. Johansen S, Cvancarova M, Ruland C (2018) The Effect of Cancer Patients’ and Their Family Caregivers’ Physical and Emotional Symptoms on Caregiver Burden. Cancer Nurs 41:91–99. https://doi.org/10.1097/NCC.0000000000000493

123. Lowery-Allison AE, Passik SD, Cribbet MR, et al (2018) Sleep problems in breast cancer survivors 1–10 years posttreatment. Palliative and Supportive Care 16:325–334. https://doi.org/10.1017/S1478951517000311

124. Overcash J, Tan A, Patel K, Noonan AM (2018) Factors Associated With Poor Sleep in Older Women Diagnosed With Breast Cancer. Oncol Nurs Forum 45:359–371. https://doi.org/10.1188/18.ONF.359-371

125. Palesh O, Scheiber C, Kesler S, et al (2018) Feasibility and acceptability of brief behavioral therapy for cancer-related insomnia: effects on insomnia and circadian rhythm during chemotherapy: a phase II randomised multicentre controlled trial. Br J Cancer 119:274–281. https://doi.org/10.1038/s41416-018-0154-2

126. Saberzadeh-Ardestani B, Khosravi B, Zebardast J, Sadighi S (2019) Chemotherapy effect on daytime sleepiness and contributing factors in older adults with cancer. J Geriatr Oncol 10:632–636. https://doi.org/10.1016/j.jgo.2018.10.003

127. Berger AM, Kupzyk KA, Djalilova DM, Cowan KH (2019) Breast Cancer Collaborative Registry Informs Understanding Of Factors Predicting Sleep Quality. Support Care Cancer 27:1365–1373. https://doi.org/10.1007/s00520-018-4417-5

128. Li W, Kwok CC-H, Chan DC-W, et al (2019) Disruption of sleep, sleep-wake activity rhythm, and nocturnal melatonin production in breast cancer patients undergoing adjuvant chemotherapy: prospective cohort study. Sleep Med 55:14–21. https://doi.org/10.1016/j.sleep.2018.11.022

129. Tejada M, Viele C, Kober KM, et al (2019) Identification of subgroups of chemotherapy patients with distinct sleep disturbance profiles and associated co-occurring symptoms. Sleep 42:. https://doi.org/10.1093/sleep/zsz151

130. Sun G-W, Yang Y-L, Yang X-B, et al (2020) Preoperative insomnia and its association with psychological factors, pain and anxiety in Chinese colorectal cancer patients. Support Care Cancer 28:2911–2919. https://doi.org/10.1007/s00520-019-05151-y

131. Papadopoulos D, Kiagia M, Charpidou A, et al (2019) Psychological correlates of sleep quality in lung cancer patients under chemotherapy: A single-center cross-sectional study. Psychooncology 28:1879–1886. https://doi.org/10.1002/pon.5167

132. Zubair UB, Mumtaz H, Taj R (2019) Relationship between the Quality of Sleep and Psychiatric Morbidity among the Patients of Head and Neck Tumours. J Coll Physicians Surg Pak 29:541–544. https://doi.org/10.29271/jcpsp.2019.06.541

133. Imanian M, Imanian M, Karimyar M (2019) Sleep Quality and Fatigue among Breast Cancer Patients Undergoing Chemotherapy. Int J Hematol Oncol Stem Cell Res 13:196–200

134. Schieber K, Niecke A, Geiser F, et al (2019) The course of cancer-related insomnia: don’t expect it to disappear after cancer treatment. Sleep Medicine 58:107–113. https://doi.org/10.1016/j.sleep.2019.02.018

135. Liou KT, Ahles TA, Garland SN, et al (2019) The Relationship Between Insomnia and Cognitive Impairment in Breast Cancer Survivors. JNCI Cancer Spectr 3:pkz041. https://doi.org/10.1093/jncics/pkz041

136. Fleming L, Randell K, Stewart E, et al (2019) Insomnia in breast cancer: a prospective observational study. Sleep 42:. https://doi.org/10.1093/sleep/zsy245

137. Fong TCT, Ho RTH (2020) Mindfulness facets predict quality of life and sleep disturbance via physical and emotional distresses in Chinese cancer patients: A moderated mediation analysis. Psycho-Oncology 29:894–901. https://doi.org/10.1002/pon.5363

138. Garland SN, Xie SX, DuHamel K, et al (2019) Acupuncture Versus Cognitive Behavioral Therapy for Insomnia in Cancer Survivors: A Randomized Clinical Trial. J Natl Cancer Inst 111:1323–1331. https://doi.org/10.1093/jnci/djz050

139. Strollo SE, Fallon EA, Gapstur SM, Smith TG (2020) Cancer-related problems, sleep quality, and sleep disturbance among long-term cancer survivors at 9-years post diagnosis. Sleep Med 65:177–185. https://doi.org/10.1016/j.sleep.2019.10.008

140. Yoshikawa K, Higashijima J, Okitsu H, et al (2020) Effects of chemotherapy on quality of life and night-time sleep of colon cancer patients. J Med Invest 67:338–342. https://doi.org/10.2152/jmi.67.338

141. Saesen K, van der Veen J, Buyse B, Nuyts S (2021) Obstructive sleep apnea in head and neck cancer survivors. Support Care Cancer 29:279–287. https://doi.org/10.1007/s00520-020-05428-7

142. İzci F, Özdem G, İlgün AS, et al (2020) Pre-Treatment and Post-Treatment Anxiety, Depression, Sleep and Sexual Function Levels in Patients with Breast Cancer. Eur J Breast Health 16:219–225. https://doi.org/10.5152/ejbh.2020.5259

143. ŞANLIER NT, ŞAHİN MA, DEMİRKAZIK A, et al (2020) Sleep Quality of Cancer Patients Receiving Systemic Treatment: A Cross-Sectional Study. Journal of Oncological Sciences 6:15–22. https://doi.org/10.37047/jos.2019-71898

144. Al Maqbali M, Hughes C, Rankin J, et al (2020) Fatigue and Sleep Disturbance in Arabic Cancer Patients After Completion of Therapy: Prevalence, Correlates, and Association With Quality of Life. Cancer Nurs 0: https://doi.org/doi: 10.1097/NCC.0000000000000825

145. Fekih-Romdhane F, Achouri L, Hakiri A, et al (2020) Hopelessness is associated with poor sleep quality after breast cancer surgery among Tunisian women. Curr Probl Cancer 44:100504. https://doi.org/10.1016/j.currproblcancer.2019.100504

146. Pai A, Sivanandh B, Udupa K (2020) Quality of Sleep in Patients with Cancer: A Cross-sectional Observational Study. Indian J Palliat Care 26:9–12. https://doi.org/10.4103/IJPC.IJPC_164_19

147. Hoang HTX, Molassiotis A, Chan CW, et al (2020) New-onset insomnia among cancer patients undergoing chemotherapy: prevalence, risk factors, and its correlation with other symptoms. Sleep Breath 24:241–251. https://doi.org/10.1007/s11325-019-01839-x

148. Chan Y-N, Jheng Y-W, Wang Y-J (2020) Chemotherapy-Induced Peripheral Neurotoxicity as A Risk Factor for Poor Sleep Quality in Breast Cancer Survivors Treated with Docetaxel. Asia Pac J Oncol Nurs 8:68–73. https://doi.org/10.4103/apjon.apjon_51_20

149. Lin C-Y, Cheng ASK, Nejati B, et al (2020) A thorough psychometric comparison between Athens Insomnia Scale and Insomnia Severity Index among patients with advanced cancer. J Sleep Res 29:e12891. https://doi.org/10.1111/jsr.12891

150. Lin C-Y, Cheng ASK, Imani V, et al (2020) Advanced psychometric testing on a clinical screening tool to evaluate insomnia: sleep condition indicator in patients with advanced cancer. Sleep Biol Rhythms 18:343–349. https://doi.org/10.1007/s41105-020-00279-5

151. Martin CM, Greene D, Harrell JP, et al (2020) The impact of social constraints on insomnia among African-American breast cancer survivors: The mediating role of fear of recurrence. Psycho-Oncology 29:1296–1302. https://doi.org/10.1002/pon.5435

152. Gonzalez BD, Eisel SL, Qin B, et al (2021) Prevalence, risk factors, and trajectories of sleep disturbance in a cohort of African-American breast cancer survivors. Support Care Cancer 29:2761–2770. https://doi.org/10.1007/s00520-020-05786-2

153. Santoso AMM, Jansen F, Lissenberg-Witte BI, et al (2021) Poor sleep quality among newly diagnosed head and neck cancer patients: prevalence and associated factors. Support Care Cancer 29:1035–1045. https://doi.org/10.1007/s00520-020-05577-9

154. Al Maqbali M (2021) Sleep Disturbance Among Arabic Breast Cancer Survivors. Support Care Cancer. https://doi.org/10.1007/s00520-021-06088-x

155. Kreutz C, Müller J, Schmidt ME, Steindorf K (2021) Comparison of subjectively and objectively assessed sleep problems in breast cancer patients starting neoadjuvant chemotherapy. Support Care Cancer 29:1015–1023. https://doi.org/10.1007/s00520-020-05580-0

156. Ratcliff CG, Zepeda SG, Hall MH, et al (2021) Patient characteristics associated with sleep disturbance in breast cancer survivors. Support Care Cancer 29:2601–2611. https://doi.org/10.1007/s00520-020-05777-3

157. Daldoul A, Ammar N, Krir MW, et al (2021) Insomnia in breast cancer: prevalence and associated factors. BMJ Support Palliat Care. https://doi.org/10.1136/bmjspcare-2020-002718

158. Nguyen NH, Vallance JK, Buman MP, et al (2021) Effects of a wearable technology-based physical activity intervention on sleep quality in breast cancer survivors: the ACTIVATE Trial. J Cancer Surviv 15:273–280. https://doi.org/10.1007/s11764-020-00930-7

159. Jeon MS, Dhillon HM, Koh E-S, et al (2021) Exploring sleep disturbance among adults with primary or secondary malignant brain tumors and their caregivers. Neurooncol Pract 8:48–59. https://doi.org/10.1093/nop/npaa057
