# Supplementary material for: Prevalence of Sleep Disturbance in Patients With Cancer: A Systematic Review and Meta-Analysis
Source: Clin Nurs Res. 2022 Apr 28;31(6):1107–23. doi: 10.1177/10547738221092146 (PMC9266067; doi:10.1177/10547738221092146)
Supplement: sj-docx-2-cnr-10.1177_10547738221092146 – Supplemental material for Prevalence of Sleep Disturbance in Patients With Cancer: A Systematic Review and Meta-Analysis [file sj-docx-2-cnr-10.1177_10547738221092146.docx]

**Table 2:** Quality assessment result of observational Studies using the Newcastle-Ottawa Scale.

|  | Study | Representativeness of the sample (One Point) | Sample Size (One Point) | Non-Respondents (One Point) | Ascertainment of the exposure (One Point) | Study controls for other variable (Two Point) | Assessment of Outcome (One Point) | Statistical Test (One Point) | Adequate Follow up time (One Point) | Score |  |
| --- | --- | --- | --- | --- | --- | --- | --- | --- | --- | --- | --- |
| 1 | (Carpenter and Andrykowski, 1998) | 0 | 0 | 1 | 1 | 1 | 1 | 1 | 0 | 5 | Moderate |
| 2 | (Savard et al., 2001) | 0 | 0 | 1 | 1 | 1 | 1 | 1 | 0 | 5 | Moderate |
| 3 | (Fortner et al., 2002) | 0 | 0 | 1 | 1 | 1 | 1 | 1 | 0 | 5 | Moderate |
| 4 | (Beck et al., 2004) | 0 | 0 | 1 | 1 | 2 | 1 | 1 | 0 | 6 | Moderate |
| 5 | (Savard et al., 2005) | 0 | 0 | 1 | 1 | 2 | 1 | 1 | 0 | 6 | Moderate |
| 6 | (Savard et al., 2005a) | 0 | 0 | 1 | 1 | 1 | 1 | 1 | 1 | 6 | Moderate |
| 7 | (Sela et al., 2005) | 0 | 0 | 0 | 1 | 2 | 1 | 1 | 0 | 5 | Moderate |
| 8 | (Carlson and Garland, 2005) | 0 | 0 | 1 | 1 | 2 | 1 | 1 | 0 | 6 | Moderate |
| 9 | (Ancoli-Israel et al., 2006) | 0 | 0 | 1 | 1 | 2 | 1 | 1 | 0 | 6 | Moderate |
| 10 | (Gooneratne et al., 2007) | 0 | 0 | 1 | 1 | 1 | 1 | 0 | 1 | 5 | Moderate |
| 11 | (Berger et al., 2007) | 0 | 0 | 1 | 1 | 2 | 1 | 1 | 1 | 7 | Low |
| 12 | (Fox et al., 2007) | 0 | 0 | 1 | 1 | 1 | 1 | 1 | 1 | 6 | Moderate |
| 13 | (Palesh et al., 2007) | 0 | 0 | 1 | 1 | 2 | 1 | 1 | 0 | 6 | Moderate |
| 14 | (Akechi et al., 2007) | 0 | 0 | 1 | 1 | 2 | 1 | 1 | 1 | 7 | Low |
| 15 | (Mystakidou et al., 2007) | 0 | 0 | 1 | 1 | 2 | 1 | 1 | 0 | 5 | Moderate |
| 16 | (Shahidi et al., 2007) | 0 | 0 | 0 | 1 | 1 | 1 | 1 | 1 | 5 | Moderate |
| 17 | (Bardwell et al., 2008) | 0 | 1 | 1 | 1 | 1 | 1 | 1 | 1 | 7 | Low |
| 18 | (Palesh et al., 2008) | 1 | 1 | 1 | 1 | 2 | 1 | 1 | 0 | 8 | Low |
| 19 | (Rogers et al., 2008) | 0 | 0 | 1 | 1 | 1 | 1 | 1 | 1 | 6 | Moderate |
| 20 | (Chen et al., 2008) | 0 | 0 | 0 | 1 | 2 | 1 | 1 | 1 | 6 | Moderate |
| 21 | (Dirksen et al., 2009) | 0 | 0 | 1 | 1 | 1 | 1 | 1 | 0 | 5 | Moderate |
| 22 | (Tremblay et al., 2009) | 0 | 0 | 1 | 1 | 1 | 1 | 1 | 1 | 6 | Moderate |
| 23 | (Liu et al., 2009) | 0 | 1 | 1 | 1 | 2 | 1 | 1 | 0 | 7 | Low |
| 24 | (Dirksen et al., 2009b) | 0 | 1 | 1 | 1 | 1 | 1 | 1 | 0 | 6 | Moderate |
| 25 | (Gibbins et al., 2009) | 0 | 0 | 1 | 1 | 1 | 1 | 1 | 0 | 5 | Moderate |
| 26 | (Mystakidou et al., 2009) | 0 | 1 | 1 | 1 | 1 | 1 | 1 | 0 | 6 | Moderate |
| 27 | (Aouizerat et al., 2009) | 0 | 0 | 1 | 1 | 2 | 1 | 1 | 1 | 7 | Low |
| 28 | (Price et al., 2009) | 0 | 0 | 1 | 1 | 2 | 1 | 1 | 1 | 7 | Low |
| 29 | (Savard et al., 2009) | 1 | 1 | 1 | 1 | 2 | 1 | 1 | 0 | 8 | Low |
| 30 | (Berger et al., 2009) | 0 | 0 | 1 | 1 | 1 | 1 | 1 | 1 | 6 | Moderate |
| 31 | (Palesh et al., 2010) | 0 | 0 | 1 | 1 | 2 | 1 | 1 | 0 | 6 | Moderate |
| 32 | (Van Onselen et al., 2010) | 1 | 0 | 1 | 1 | 1 | 1 | 1 | 0 | 6 | Moderate |
| 33 | (Vargas et al., 2010) | 0 | 1 | 1 | 1 | 1 | 1 | 1 | 0 | 6 | Moderate |
| 34 | (Otte et al., 2010) | 1 | 0 | 1 | 1 | 1 | 1 | 1 | 0 | 6 | Moderate |
| 35 | (Dodd et al., 2010) | 0 | 0 | 1 | 1 | 2 | 1 | 1 | 0 | 6 | Moderate |
| 36 | (Barsevick et al., 2010) | 0 | 0 | 1 | 1 | 1 | 1 | 1 | 1 | 6 | Moderate |
| 37 | (Beck et al., 2010) | 0 | 0 | 1 | 1 | 1 | 1 | 1 | 1 | 6 | Moderate |
| 38 | (Park et al., 2010) | 0 | 0 | 1 | 1 | 1 | 1 | 1 | 1 | 6 | Moderate |
| 39 | (Garrett et al., 2011) | 0 | 0 | 1 | 1 | 2 | 1 | 1 | 0 | 5 | Moderate |
| 40 | (Miaskowski et al., 2011) | 0 | 0 | 1 | 1 | 2 | 1 | 1 | 1 | 7 | Low |
| 41 | (Miaskowski et al., 2011a) | 1 | 0 | 1 | 1 | 1 | 1 | 1 | 0 | 6 | Moderate |
| 42 | (Sandadi et al., 2011) | 0 | 0 | 1 | 1 | 1 | 1 | 1 | 0 | 5 | Moderate |
| 43 | (Delgado-Guay et al., 2011) | 0 | 0 | 1 | 1 | 1 | 1 | 1 | 1 | 6 | Moderate |
| 44 | (Bower et al., 2011) | 0 | 0 | 1 | 1 | 1 | 1 | 1 | 1 | 6 | Moderate |
| 45 | (Hanisch et al., 2011) | 0 | 1 | 1 | 1 | 1 | 1 | 1 | 0 | 6 | Moderate |
| 46 | (Colagiuri et al., 2011) | 0 | 1 | 1 | 1 | 2 | 1 | 1 | 1 | 8 | Low |
| 47 | (Moore et al., 2011) | 0 | 1 | 1 | 1 | 1 | 1 | 1 | 0 | 6 | Moderate |
| 48 | (Grutsch et al., 2011) | 0 | 0 | 1 | 1 | 2 | 1 | 1 | 0 | 6 | Moderate |
| 49 | (Sun et al., 2011) | 0 | 0 | 1 | 1 | 1 | 1 | 1 | 1 | 6 | Moderate |
| 50 | (Rand et al., 2011) | 0 | 0 | 0 | 1 | 1 | 1 | 1 | 1 | 5 | Moderate |
| 51 | (Kotronoulas et al., 2011) | 0 | 0 | 1 | 1 | 1 | 1 | 1 | 1 | 6 | Moderate |
| 52 | (Enderlin et al., 2011) | 0 | 0 | 0 | 1 | 1 | 1 | 1 | 1 | 5 | Moderate |
| 53 | (Dhruva et al., 2012) | 0 | 1 | 1 | 1 | 1 | 1 | 1 | 0 | 6 | Moderate |
| 54 | (Phillips et al., 2012) | 0 | 0 | 1 | 1 | 1 | 1 | 1 | 0 | 5 | Moderate |
| 55 | (Forsythe et al., 2012) | 0 | 0 | 1 | 1 | 1 | 1 | 1 | 1 | 6 | Moderate |
| 56 | (Liu et al., 2012) | 0 | 0 | 1 | 1 | 2 | 1 | 1 | 1 | 7 | Low |
| 57 | (Mosher and Duhamel, 2012) | 0 | 1 | 1 | 1 | 2 | 1 | 1 | 0 | 7 | Low |
| 58 | (Liu et al., 2012a) | 0 | 1 | 1 | 1 | 1 | 1 | 1 | 0 | 6 | Moderate |
| 59 | (Clevenger et al., 2012) | 0 | 0 | 1 | 1 | 2 | 1 | 1 | 0 | 6 | Moderate |
| 60 | (Van Onselen et al., 2012) | 0 | 1 | 1 | 1 | 2 | 1 | 1 | 0 | 7 | Low |
| 61 | (Mansano-Schlosser and Ceolim, 2012) | 0 | 0 | 0 | 1 | 1 | 1 | 1 | 1 | 5 | Moderate |
| 62 | (Taylor et al., 2012) | 0 | 0 | 0 | 1 | 1 | 1 | 1 | 1 | 5 | Moderate |
| 63 | (Tzeng et al., 2012) | 0 | 0 | 1 | 1 | 1 | 1 | 1 | 1 | 6 | Moderate |
| 64 | (Desai et al., 2013) | 0 | 0 | 1 | 1 | 1 | 1 | 1 | 0 | 5 | Moderate |
| 65 | (Clevenger et al., 2013) | 0 | 0 | 1 | 1 | 2 | 1 | 1 | 0 | 6 | Moderate |
| 66 | (Sanford et al., 2013) | 0 | 0 | 1 | 1 | 1 | 1 | 1 | 1 | 6 | Moderate |
| 67 | (Saini et al., 2013) | 0 | 0 | 0 | 1 | 2 | 1 | 1 | 0 | 5 | Moderate |
| 68 | (Caplette-Gingras et al., 2013) | 0 | 0 | 0 | 1 | 1 | 1 | 1 | 1 | 5 | Moderate |
| 69 | (Nakamura et al., 2013) | 0 | 0 | 1 | 1 | 1 | 1 | 1 | 1 | 6 | Moderate |
| 70 | (Hall et al., 2014) | 0 | 0 | 1 | 1 | 1 | 0 | 1 | 1 | 5 | Moderate |
| 71 | (Ho and Fong, 2014) | 0 | 0 | 1 | 1 | 2 | 1 | 1 | 1 | 7 | Low |
| 72 | (Ma et al., 2014) | 0 | 0 | 1 | 1 | 2 | 1 | 1 | 0 | 6 | Moderate |
| 73 | (Hong et al., 2014) | 0 | 0 | 0 | 1 | 2 | 1 | 1 | 0 | 5 | Moderate |
| 74 | (Mao et al., 2014) | 0 | 0 | 1 | 1 | 2 | 1 | 1 | 0 | 6 | Moderate |
| 75 | (Romito et al., 2014) | 0 | 1 | 1 | 1 | 2 | 1 | 1 | 0 | 7 | Low |
| 76 | (Vargas et al., 2014) | 1 | 1 | 1 | 1 | 2 | 1 | 1 | 0 | 8 | Low |
| 77 | (Courneya et al., 2014) | 0 | 1 | 1 | 1 | 2 | 1 | 1 | 0 | 7 | Low |
| 78 | (Kashani and Kashani, 2014) | 0 | 0 | 1 | 1 | 1 | 1 | 1 | 1 | 6 | Moderate |
| 79 | (Valko et al., 2015) | 1 | 0 | 1 | 1 | 1 | 1 | 1 | 0 | 6 | Moderate |
| 80 | (Ho et al., 2015) | 0 | 0 | 1 | 1 | 2 | 1 | 1 | 0 | 6 | Moderate |
| 81 | (Klyushnenkova et al., 2015) | 0 | 0 | 1 | 1 | 1 | 1 | 1 | 0 | 5 | Moderate |
| 82 | (Mercadante et al., 2015) | 0 | 1 | 1 | 1 | 1 | 1 | 1 | 1 | 7 | Low |
| 83 | (He et al., 2015) | 0 | 0 | 1 | 1 | 1 | 1 | 1 | 0 | 5 | Moderate |
| 84 | (Nishiura et al., 2015) | 0 | 1 | 1 | 1 | 1 | 1 | 1 | 0 | 6 | Moderate |
| 85 | (Doong et al., 2015) | 0 | 0 | 1 | 1 | 1 | 1 | 1 | 1 | 6 | Moderate |
| 86 | (Yu and Nho, 2015) | 0 | 0 | 0 | 1 | 2 | 1 | 1 | 0 | 5 | Moderate |
| 87 | (Qin et al., 2015) | 0 | 0 | 0 | 1 | 2 | 1 | 1 | 0 | 5 | Moderate |
| 88 | (Morris et al., 2015) | 0 | 1 | 1 | 1 | 2 | 1 | 1 | 0 | 7 | Low |
| 89 | (Tian et al., 2015) | 0 | 0 | 1 | 1 | 2 | 1 | 1 | 0 | 6 | Moderate |
| 90 | (Savard et al., 2015) | 0 | 1 | 1 | 1 | 2 | 1 | 1 | 0 | 7 | Low |
| 91 | (Savard et al., 2015) | 0 | 1 | 1 | 1 | 2 | 1 | 1 | 0 | 7 | Low |
| 92 | (Akman et al., 2015) | 0 | 0 | 1 | 1 | 1 | 1 | 1 | 1 | 6 | Moderate |
| 93 | (Berrett-Abebe et al., 2015) | 0 | 0 | 0 | 1 | 1 | 1 | 1 | 1 | 5 | Moderate |
| 94 | (Roscoe et al., 2015) | 0 | 1 | 1 | 1 | 2 | 1 | 1 | 0 | 7 | Low |
| 95 | (Lafçi and Öztunç, 2015) | 0 | 0 | 0 | 1 | 1 | 1 | 1 | 1 | 5 | Moderate |
| 96 | (Jung et al., 2016) | 0 | 0 | 1 | 1 | 1 | 1 | 1 | 0 | 5 | Moderate |
| 97 | (Bagheri-Nesami et al., 2016) | 0 | 0 | 1 | 1 | 1 | 1 | 1 | 0 | 5 | Moderate |
| 98 | (Li et al., 2017) | 0 | 0 | 1 | 1 | 1 | 0 | 1 | 1 | 5 | Moderate |
| 99 | (Collins et al., 2017) | 1 | 0 | 1 | 1 | 1 | 1 | 1 | 0 | 6 | Moderate |
| 100 | (Fontes et al., 2017) | 0 | 0 | 1 | 1 | 2 | 1 | 1 | 1 | 7 | Low |
| 101 | (Loth et al., 2017) | 0 | 0 | 1 | 1 | 1 | 1 | 1 | 0 | 5 | Moderate |
| 102 | (Mercadante et al., 2017) | 0 | 0 | 1 | 1 | 1 | 1 | 1 | 1 | 6 | Moderate |
| 103 | (Halle et al., 2017) | 0 | 0 | 1 | 1 | 2 | 1 | 0 | 1 | 6 | Moderate |
| 104 | (Chung et al., 2017) | 0 | 0 | 0 | 1 | 1 | 1 | 1 | 1 | 5 | Moderate |
| 105 | (Ji et al., 2017) | 0 | 0 | 0 | 1 | 1 | 1 | 1 | 1 | 5 | Moderate |
| 106 | (Davies et al., 2017) | 0 | 0 | 1 | 1 | 1 | 1 | 1 | 1 | 6 | Moderate |
| 107 | (Echchikhi et al., 2017) | 0 | 0 | 0 | 1 | 1 | 1 | 1 | 1 | 5 | Moderate |
| 108 | (Rogers et al., 2017) | 0 | 1 | 1 | 1 | 2 | 1 | 1 | 0 | 7 | Low |
| 109 | (Cha et al., 2017) | 0 | 0 | 0 | 1 | 1 | 1 | 1 | 1 | 5 | Moderate |
| 110 | (Peoples et al., 2017) | 0 | 0 | 0 | 1 | 2 | 1 | 1 | 0 | 5 | Moderate |
| 111 | (Mansano-Schlosser et al., 2017) | 0 | 0 | 1 | 1 | 1 | 1 | 1 | 1 | 6 | Moderate |
| 112 | (Li et al., 2017b) | 0 | 0 | 1 | 1 | 1 | 1 | 1 | 1 | 6 | Moderate |
| 113 | (Miladinia et al., 2018) | 0 | 0 | 1 | 1 | 1 | 1 | 1 | 1 | 6 | Moderate |
| 114 | (Gu et al., 2018) | 0 | 0 | 1 | 1 | 1 | 1 | 1 | 0 | 5 | Moderate |
| 115 | (Adams et al., 2018) | 0 | 0 | 1 | 1 | 2 | 1 | 1 | 0 | 6 | Moderate |
| 116 | (Steel et al., 2018) | 1 | 0 | 1 | 1 | 1 | 1 | 1 | 0 | 6 | Moderate |
| 117 | (Henneghan et al., 2018) | 0 | 0 | 1 | 1 | 1 | 1 | 1 | 0 | 5 | Moderate |
| 118 | (Jim et al., 2018) | 0 | 0 | 1 | 1 | 2 | 1 | 1 | 0 | 6 | Moderate |
| 119 | (Nelson et al., 2018) | 0 | 0 | 1 | 1 | 1 | 1 | 1 | 0 | 5 | Moderate |
| 120 | (Gonzalez et al., 2018) | 0 | 0 | 1 | 1 | 1 | 1 | 1 | 1 | 6 | Moderate |
| 121 | (Dreher et al., 2018) | 0 | 0 | 1 | 1 | 1 | 1 | 1 | 0 | 5 | Moderate |
| 122 | (Mao et al., 2018) | 1 | 1 | 1 | 1 | 2 | 1 | 1 | 0 | 8 | Low |
| 123 | (Johansen et al., 2018) | 0 | 0 | 1 | 1 | 1 | 1 | 1 | 1 | 6 | Moderate |
| 124 | (Lowery-Allison et al., 2018) | 0 | 0 | 0 | 1 | 1 | 1 | 1 | 1 | 5 | Moderate |
| 125 | (Overcash et al., 2018) | 0 | 0 | 0 | 1 | 1 | 1 | 1 | 1 | 5 | Moderate |
| 126 | (Palesh et al., 2018) | 0 | 0 | 1 | 1 | 1 | 1 | 1 | 1 | 6 | Moderate |
| 127 | (Saberzadeh-Ardestani et al., 2019) | 0 | 0 | 1 | 1 | 2 | 1 | 0 | 0 | 5 | Moderate |
| 128 | (Berger et al., 2019) | 0 | 1 | 1 | 1 | 2 | 1 | 1 | 0 | 7 | Low |
| 129 | (Li et al., 2019) | 0 | 0 | 1 | 1 | 1 | 1 | 1 | 0 | 5 | Moderate |
| 130 | (Tejada et al., 2019) | 0 | 1 | 1 | 1 | 2 | 1 | 1 | 0 | 7 | Low |
| 131 | (Papadopoulos et al., 2019) | 0 | 0 | 1 | 1 | 1 | 1 | 1 | 0 | 5 | Moderate |
| 132 | (Zubair et al., 2019) | 0 | 0 | 1 | 1 | 1 | 1 | 1 | 0 | 5 | Moderate |
| 133 | (Imanian et al., 2019) | 0 | 0 | 1 | 1 | 1 | 1 | 1 | 0 | 5 | Moderate |
| 134 | (Schieber et al., 2019) | 0 | 0 | 0 | 1 | 1 | 1 | 1 | 1 | 5 | Moderate |
| 135 | (Liou et al., 2019) | 0 | 0 | 1 | 1 | 2 | 1 | 1 | 1 | 7 | Low |
| 136 | (Fleming et al., 2019) | 0 | 1 | 1 | 1 | 2 | 1 | 1 | 0 | 7 | Low |
| 137 | (Garland et al., 2019) | 0 | 0 | 1 | 1 | 1 | 1 | 1 | 1 | 6 | Moderate |
| 138 | (Sun et al., 2020) | 0 | 0 | 1 | 1 | 1 | 1 | 1 | 1 | 6 | Moderate |
| 139 | (Strollo et al., 2020) | 0 | 0 | 1 | 1 | 1 | 1 | 1 | 0 | 5 | Moderate |
| 140 | (Yoshikawa et al., 2020) | 0 | 0 | 1 | 1 | 1 | 1 | 1 | 0 | 5 | Moderate |
| 141 | (İzci et al., 2020) | 0 | 0 | 1 | 1 | 1 | 1 | 1 | 0 | 5 | Moderate |
| 142 | (ŞANLIER et al., 2020) | 1 | 0 | 1 | 1 | 1 | 1 | 1 | 0 | 6 | Moderate |
| 143 | (Al Maqbali et al., 2020) | 0 | 0 | 1 | 1 | 2 | 1 | 1 | 0 | 6 | Moderate |
| 144 | (Fekih-Romdhane et al., 2020) | 0 | 0 | 0 | 1 | 1 | 1 | 1 | 1 | 5 | Moderate |
| 145 | (Pai et al., 2020) | 0 | 0 | 1 | 1 | 1 | 1 | 1 | 0 | 5 | Moderate |
| 146 | (Fong and Ho, 2020) | 0 | 0 | 1 | 1 | 2 | 1 | 1 | 0 | 6 | Moderate |
| 147 | (Hoang et al., 2020) | 0 | 0 | 0 | 1 | 1 | 1 | 1 | 1 | 5 | Moderate |
| 148 | (Chan et al., 2020) | 0 | 0 | 0 | 1 | 1 | 1 | 1 | 1 | 5 | Moderate |
| 149 | (Lin et al., 2020) | 0 | 0 | 1 | 1 | 2 | 1 | 1 | 0 | 6 | Moderate |
| 150 | (Lin et al., 2020a) | 1 | 1 | 1 | 1 | 2 | 1 | 1 | 0 | 8 | Low |
| 151 | (Martin et al., 2020) | 0 | 0 | 1 | 1 | 1 | 1 | 1 | 1 | 6 | Moderate |
| 152 | (Saesen et al., 2021) | 0 | 0 | 0 | 1 | 2 | 1 | 1 | 0 | 5 | Moderate |
| 153 | (Gonzalez et al., 2021) | 0 | 0 | 1 | 1 | 2 | 1 | 1 | 0 | 6 | Moderate |
| 154 | (Santoso et al., 2021) | 0 | 0 | 1 | 1 | 2 | 1 | 1 | 0 | 6 | Moderate |
| 155 | (Al Maqbali, 2021) | 0 | 0 | 1 | 1 | 1 | 1 | 1 | 0 | 5 | Moderate |
| 156 | (Kreutz et al., 2021) | 0 | 0 | 0 | 1 | 2 | 1 | 1 | 1 | 6 | Moderate |
| 157 | (Ratcliff et al., 2021) | 0 | 0 | 1 | 1 | 2 | 1 | 1 | 0 | 6 | Moderate |
| 158 | (Daldoul et al., 2021) | 0 | 0 | 0 | 1 | 1 | 1 | 1 | 1 | 5 | Moderate |
| 159 | (Nguyen et al., 2021) | 0 | 0 | 1 | 1 | 1 | 1 | 1 | 1 | 6 | Moderate |
| 160 | (Jeon et al., 2021) | 0 | 0 | 1 | 1 | 1 | 1 | 1 | 0 | 5 | Moderate |

Ratings: Low Risk of Bias 7-9: Moderate Risk of Bias: 5-6: High Risk of Bias: 1-4.
